# Gut Microbiome–Brain Alliance: A Landscape View into Mental and Gastrointestinal Health and Disorders

**DOI:** 10.1021/acschemneuro.3c00127

**Published:** 2023-05-08

**Authors:** Janet M. Sasso, Ramy M. Ammar, Rumiana Tenchov, Steven Lemmel, Olaf Kelber, Malte Grieswelle, Qiongqiong Angela Zhou

**Affiliations:** † CAS, a division of the American Chemical Society, 2540 Olentangy River Rd, Columbus, Ohio 43202, United States; ‡ Bayer Consumer Health, R&D Digestive Health, Darmstadt 64295, Germany

**Keywords:** gut, intestine, microorganism, bacteria, microbiota, brain, DGBI, metabolite, mental, dysbiosis

## Abstract

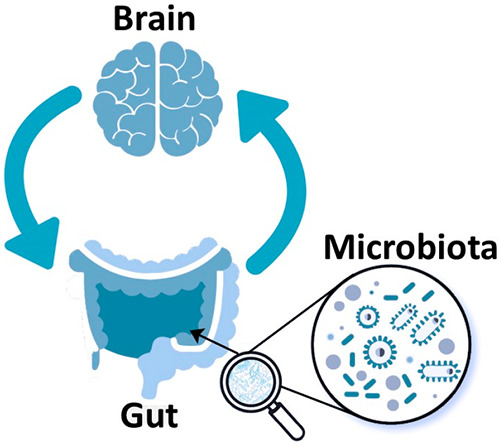

Gut microbiota includes a vast collection of microorganisms residing within the gastrointestinal tract. It is broadly recognized that the gut and brain are in constant bidirectional communication, of which gut microbiota and its metabolic production are a major component, and form the so-called gut microbiome–brain axis. Disturbances of microbiota homeostasis caused by imbalance in their functional composition and metabolic activities, known as dysbiosis, cause dysregulation of these pathways and trigger changes in the blood–brain barrier permeability, thereby causing pathological malfunctions, including neurological and functional gastrointestinal disorders. In turn, the brain can affect the structure and function of gut microbiota through the autonomic nervous system by regulating gut motility, intestinal transit and secretion, and gut permeability. Here, we examine data from the CAS Content Collection, the largest collection of published scientific information, and analyze the publication landscape of recent research. We review the advances in knowledge related to the human gut microbiome, its complexity and functionality, its communication with the central nervous system, and the effect of the gut microbiome–brain axis on mental and gut health. We discuss correlations between gut microbiota composition and various diseases, specifically gastrointestinal and mental disorders. We also explore gut microbiota metabolites with regard to their impact on the brain and gut function and associated diseases. Finally, we assess clinical applications of gut-microbiota-related substances and metabolites with their development pipelines. We hope this review can serve as a useful resource in understanding the current knowledge on this emerging field in an effort to further solving of the remaining challenges and fulfilling its potential.

## Introduction

The Earth microbiome represents the majority of the planet’s biodiversity. Microbial life was the first to inhabit Earth.^1^ Microbes regulate global nutrient cycles, greenhouse gas exchange, as well as disease transmission and protection, thus providing essential life support to the planet.^2^ Among many other harbors, including plants, animals, soil, and entire ecosystems, a wide diversity of microorganisms colonize the human body, which are now known to play an essential role in the human host by regulating key physiological functions.

The large collection of microorganisms inhabiting the human body are predominantly bacteria, but also viruses, protozoa, fungi, and archaea. They are collectively known as the human microbiota. Those microorganisms residing in the digestive tracts are known as gut flora or gut microbiota. As a matter of fact, there are more bacterial cells in the human body than human cells—roughly 40 trillion bacterial cells versus only 30 trillion human cells. Together, they function as an extra organ in the human body—a so-called “forgotten organ”—since these microbes have a collective metabolic activity equal to a virtual organ.^3^ The collective genome of the gut microbes, the gut microbiome, exceeds over 100 times the amount of human genome in the body.^4^ Considering such enormous genetic potential of the microbiota, it is anticipated that it plays a role in virtually all physiological processes in the human body, including metabolic functions and immune homeostasis.^5−11^

**Figure 1:**
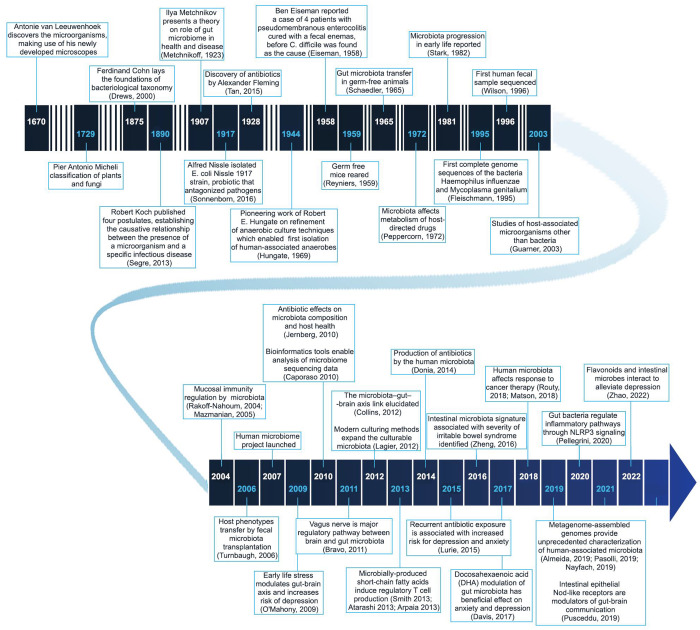
Timeline of major research and development milestones related to the microbiome.^12−30,39,49−80^

Despite being considered a relatively new field of research, the first reports of human-associated microbiota date back to the 17th century when Antonie van Leeuwenhoek described five different kinds of oral bacteria.^12^ In the following decades, the foundations of microbiology were laid, and knowledge of the host–microorganism interactions has accumulated (Figure 1).^13−22^ Despite these early findings, rapid development of the field only started when methods to culture anaerobic organisms were set up in the mid-20th century when representatives of the microbiota were grown and studied in the laboratory.^23^

In the 2010s, the gut microbiome field burst into the life sciences research and industry, which prompted Forbes to declare the 2010s “The Decade of the Microbiome.”^24^ This growth in the field was largely related to the National Institutes of Health’s “The Human Microbiome Project”^25^ and the MetaHIT project funded by the European Union.^26^ In 2005, the International Human Microbiome Consortium (IHMC) was founded in a cooperative effort to study the microbiome in human health and disease with the ultimate goal of applying this knowledge to prevent and/or treat diseases, and the abovementioned megaprojects have contributed to fulfilling this goal. They provided significant evidence for the relationship between metabolic, neurological, and autoimmune disorders; allergies, infections, and cancers; and the microorganisms that live on and in humans. Specifically, gastrointestinal diseases/disorders, such as inflammatory bowel diseases that include both Crohn’s disease and ulcerative colitis, irritable bowel syndrome (IBS), functional dyspepsia (FD), constipation, celiac disease, and more, are attracting attention with their close relation to the gut microbiome. Because of these findings and the essential part the gut microbiome plays in drug metabolism, the microbiome has become a popular target in the biotechnology industry. In 2010, the first extensive catalogue of human intestinal microbial genes was published on the basis of the studies of 124 individuals.^27^ In 2011, the Human Microbiome Project published the sequences of 178 bacterial species.^28^

Although DNA sequencing has been used for decades, it was only after the development of next-generation sequencing when metagenomic studies became affordable.^29^ The term metagenomics is used to describe genetic studies of microbial assemblies from environmental samples using sequence-based bioinformatics tools.^30^ The goal of these studies is to identify the taxonomic diversity of the microbiota and to differentiate the biological roles of the representatives of such samples by performing functional metagenomics.

The human microbiota plays an essential role in human physiology and pathology. It collaborates closely with the digestive tract in several important aspects: (a) it promotes digestion by assisting the absorption of nutrients by gut cells or the fermentation of some food fractions, which generate important metabolites, including short-chain fatty acids;^31^ (b) it supports the maturation of the digestive tract by participating in the assembly of gastrointestinal mucus and promoting the enzymatic activity of the mucosa;^32^ (c) it performs a barrier function against pathogens and toxins, where some bacteria release antimicrobial agents that protect from the pathogenic bacteria;^33^ (d) it plays a protective role in promoting the immune system development; and (e) it supports in the synthesis of essential vitamins like vitamin B: Magnúsdóttir et al. estimated that 86% of the recommended daily allowance (RDA) of vitamin B6, 37% of the RDA of vitamin B9, 31% of the RDA of vitamin B12, and 27% of the RDA of vitamin B3 could be provided by the human gut microbiota.^34^

Gut microbial disruption (dysbiosis) causes not only gastrointestinal disorders but also disorders in other distal organs and systems. Not long ago, it was found that gut bacteria can affect the central nervous system (CNS) functions.^35−38^ Indeed, the gut and brain are in constant bidirectional communication, of which the microbiota and its metabolic production are a major component. The gut and brain connect via a neuro-immuno-humoral network of signaling pathways known as the gut microbiome–brain axis, which includes the vagus nerve, the immune system, the hormonal system, and bacterial metabolites and products.^39^ The digestive system, including the inhabiting microbiota, was even called “the second brain”^40^ at the time when scientists were beginning to realize that the gut and the brain in humans were involved in constant crosstalk and significantly modulate each other’s function. During disturbance of the microbiota homeostasis caused by an imbalance in their functional composition and metabolic activities, known as dysbiosis, these routes are dysregulated and cause changes in the permeability of the blood–brain barrier (BBB), neuroinflammation, and other pathological malfunctions, including a range of neurodevelopmental and neurodegenerative disorders.^41^ Disorders of the gut–brain interaction (DGBI) is the recent term proposed by Rome Foundation guidelines for a range of functional gastrointestinal disorders including but not limited to IBS, FD, and functional constipation. This highlights the central role of the miscommunication between the gut and brain in these digestive disorders.^42^ The gut microorganisms transform and metabolize dietary- and host-derived substances to generate a diverse set of metabolites with important local and systemic outcomes, thereby building a network of immunological, neuronal, and endocrine signaling pathways.

It is generally believed that bacterial colonization begins during birth.^43^ The neonatal microbiota differs depending on mode of delivery: in vaginally delivered infants, it resembles the maternal vaginal microbiota, while the microbiota in those delivered by cesarean section resembles the maternal skin microbiota.^44^ Premature birth, feeding method, and perinatal administration of antibiotics are also among the conditions affecting the development of the neonatal microbiome.^45^ Recently, a new mode of horizontal mother-to-infant microbiome transmission has been revealed where microbes in the maternal gut shared genes with microbes in the infant gut during the perinatal period, which starts shortly before birth and prolongs throughout the first few weeks after birth.^46^ A major factor of gut microbiota composition during adulthood is diet. Prompt changes in microbiota composition take place in response to dietary style changes. Specific patterns have been reported in plant-based versus animal-based diets.^47,48^ The development and modifications of the gut microbiota are influenced by multiple other factors, such as exposure to stress, environmental conditions, medications intake, lifecycle, medical disorders, and procedures.

The human gut microbiota is divided into many groups called phyla. The gut microbiota primarily comprises four main phyla, including Firmicutes, Bacteroidetes, Actinobacteria, and Proteobacteria, with the Firmicutes and Bacteroidetes representing 90% of gut microbiota.^81,82^ The majority of bacteria reside within the gastrointestinal tract, with most predominantly anaerobic bacteria housed in the large intestine.^83^

In recent years, sizable technological progress and wealth of knowledge have promoted the advancement of microbiome research, thereby enhancing our understanding of its relationship to human physiology and pathologies. In this paper, we review the advances in the knowledge related to the human gut microbiome, its complexity and functionality, its communication with the central nervous system, and the effect of the gut microbiome–brain axis on mental and digestive health. We examine data from the CAS Content Collection,^15^ the largest human-curated collection of published scientific information and analyze the publication landscape of recent research in order to provide insights into the scientific advances in the area. We also discuss the correlations between the gut microbiota composition and various diseases, specifically digestive system diseases, mental, and neurodegenerative disorders. We furthermore explore the gut microbiota metabolites with regard to their impact on brain, digestive functions, and their associated diseases. Subsequently, we assess the clinical applications of gut microbiota-related substances and metabolites, their development pipelines, disease categories, development stages, and publication trends. We hope this review can serve as a useful resource in understanding the current state of knowledge in the field of gut microbes and the gut microbiome–brain interactions in an effort to further solve the remaining challenges for fulfilling the potential of the field.

## Landscape of Gut Microbiome Research—Insights from the CAS Content Collection

**Figure 2:**
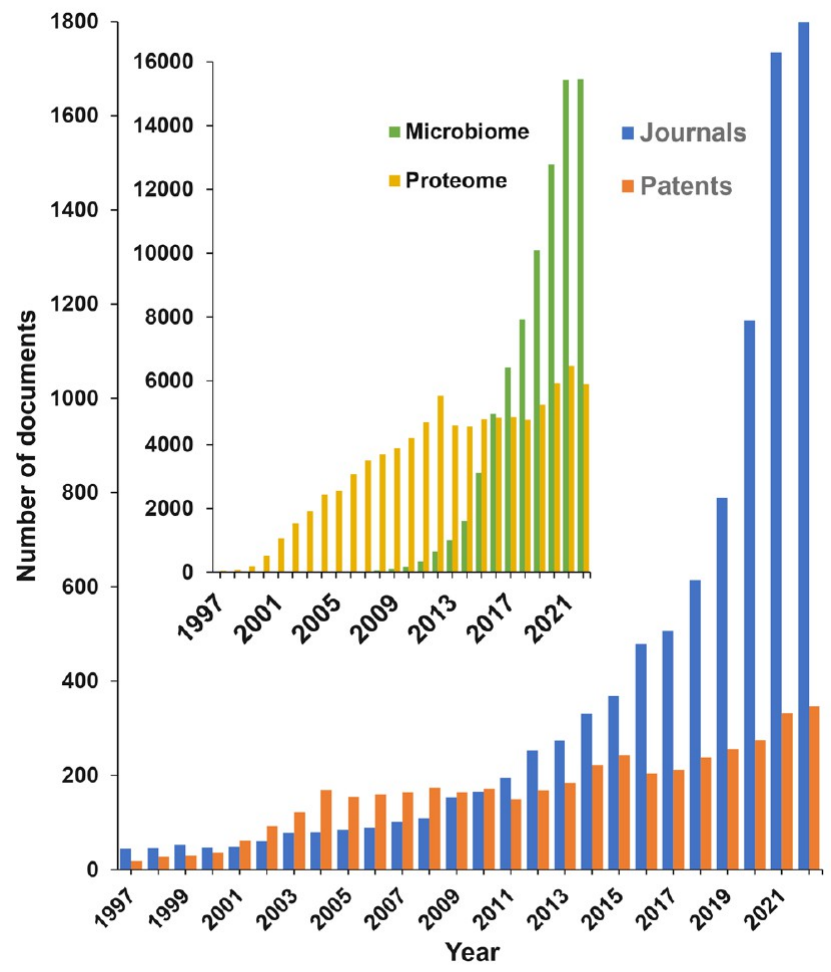
Journal and patent publication trends on gut microbiome research related to mental and gut health according to the CAS Content Collection. Inset: microbiome vs proteome document yearly trends.

The CAS Content Collection^84^ is the largest human-curated collection of published scientific knowledge. It is a comprehensive resource to access and remain well-informed on the world’s available scientific literature across disciplines, including chemistry, biomedical sciences, engineering, materials science, agricultural science, and many more, thus allowing quantitative analysis of global scientific publications against variables, such as time, research area, application, disease association, and chemical composition. A search in the CAS Content Collection showed an intense increase of the documents related to microbiome research in the past decade, which overcame other “omics” exploration—for example, the number of proteomics-related documents held up after the initial burst in the early 2000s and were surpassed by the microbiome documents after 2016 (Figure 2, inset). Currently, there are over 250 000 scientific publications (mainly journal articles and patents) in the CAS Content Collection related to gut/intestinal microbiome/microbiota. Nearly 15 000 of them are related to various aspects of mental and gut health. There is a steady, exponential growth of the number of journal articles over time that has been rather explosive from 2021–2022 (Figure 2). The number of patents rapidly grew until 2004, which possibly correlated with the initial accumulation of knowledge and its transfer into patentable applications. Later on, the growth substantially slowed down, perhaps awaiting the forthcoming breakthroughs in the gut microbiome awareness (Figure 2).

**Figure 3:**
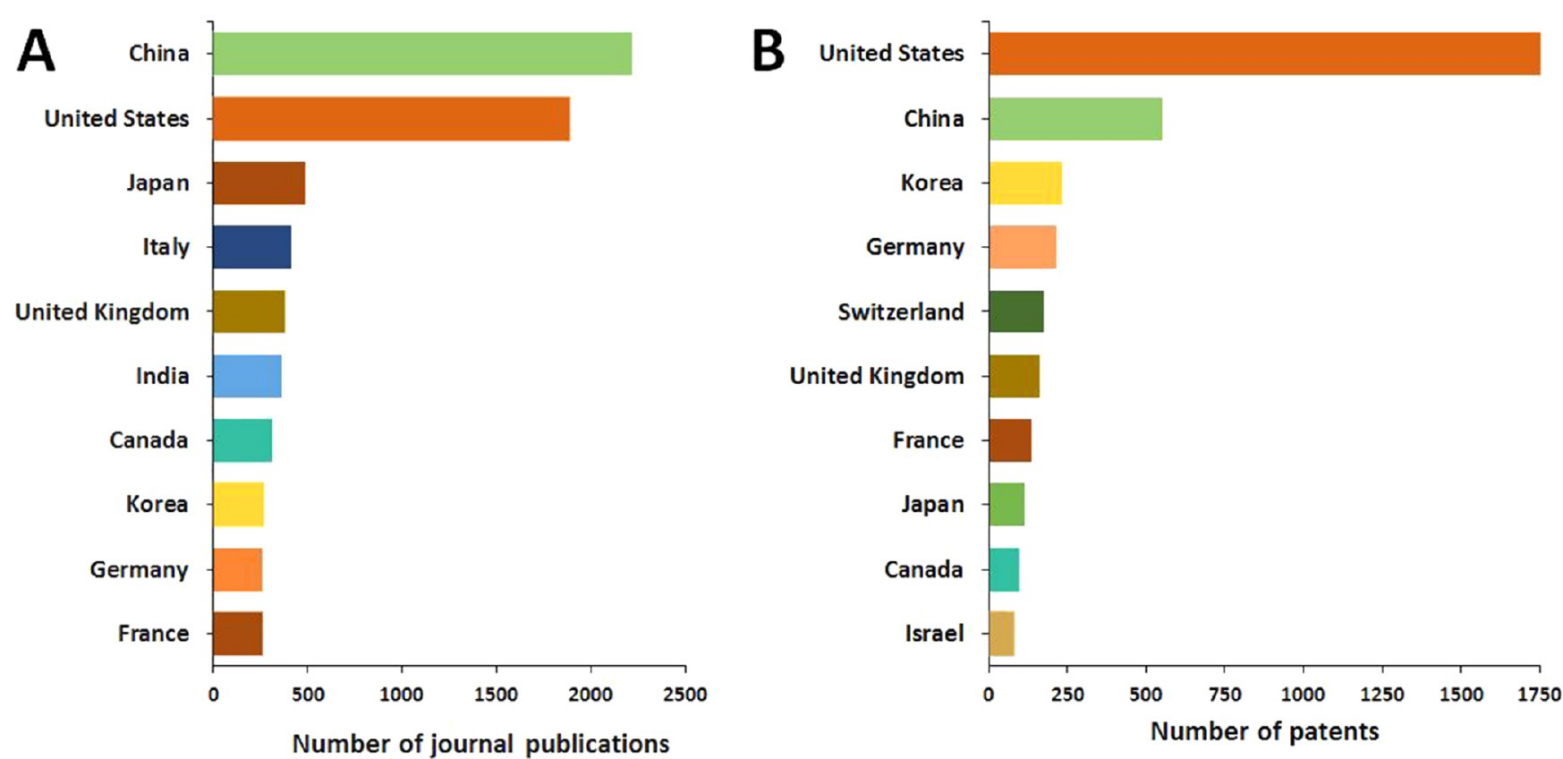
Top countries publishing journal articles (A) and patents (B) related to gut microbiome research in mental and gut health.

The United States, China, Japan, and Korea are the leaders in the number of published journal articles (Figure 3A) and patents (Figure 3B) related to gut microbiome research in the areas of mental and gut health.

**Figure 4:**
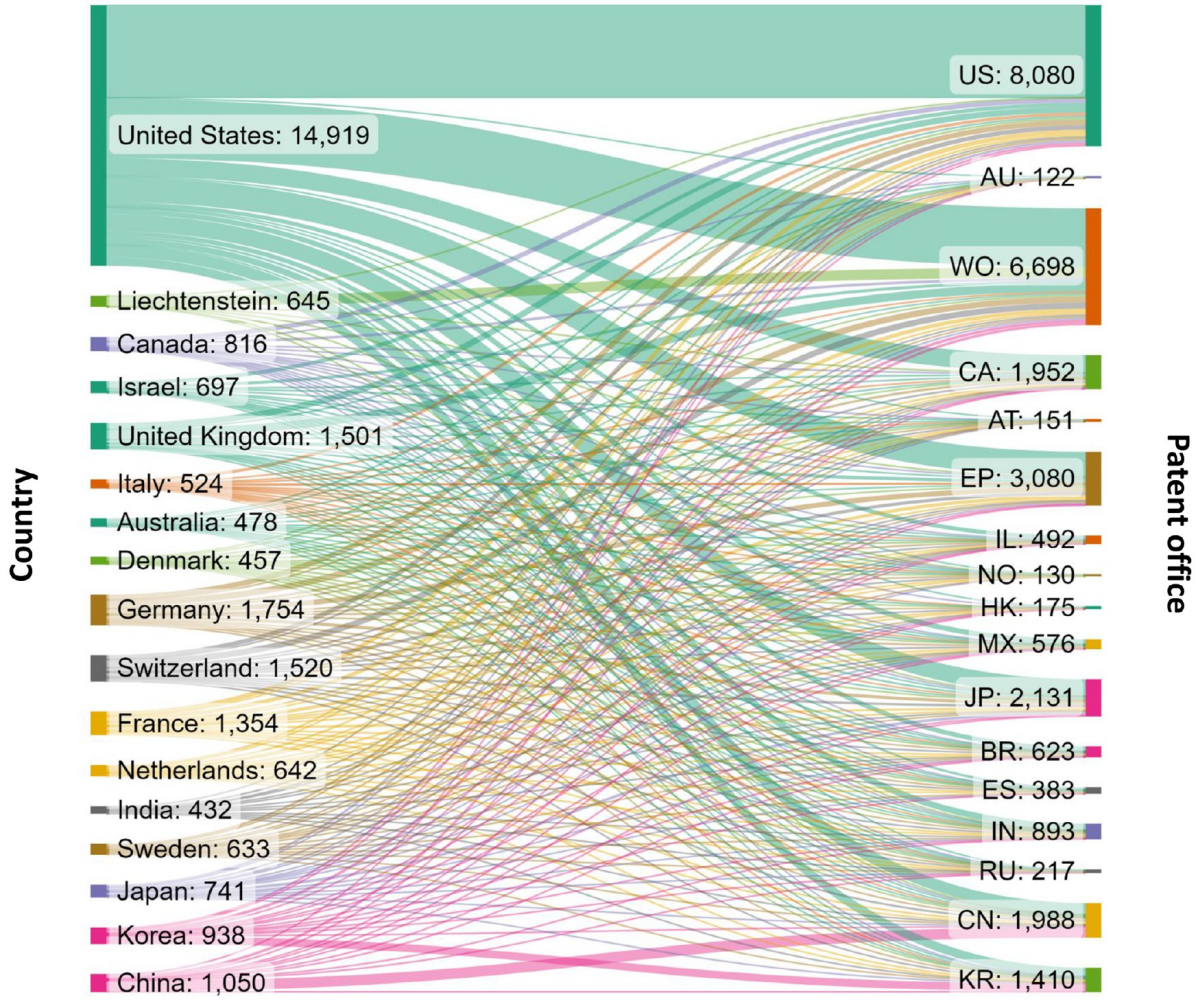
Flow of patent filings related to gut microbiome research in mental and gut health from different patent assignee locations (left) to various patent offices of filing (right). The abbreviations on the right indicate the patent offices of United States (US), Australia (AU), World Intellectual Property Organization (WO), Canada (CA), Austria (AT), European Patent Office (EP), Israel (IL), Norway (NO), Hong Kong (HK), Mexico (MX), Japan (JP), Brazil (BR), Spain (ES), India (IN), Russian Federation (RU), China (CN), and Korea (KR).

Figure 4 presents the flow of patent filings from different applicant locations to various patent offices. Because patent protection is territorial, and the same invention may be filed for protection in multiple jurisdictions, we looked at all relevant filings on gut microbiome research in mental and gut health. One patent family may have been counted multiple times when it was applied in multiple patent offices. There are diverse patent filing strategies: some patent assignees, such as those from China and Korea, file foremost in their home country patent offices (CN, KR), with a smaller proportion filing through other patent offices or other jurisdictions. Others, for instance United States-based applicants, have a nearly equal number of US and WO filings and a considerable number of filings at other patent offices, such as the European Patent Office (EP) and Canada (CA).

**Figure 5:**
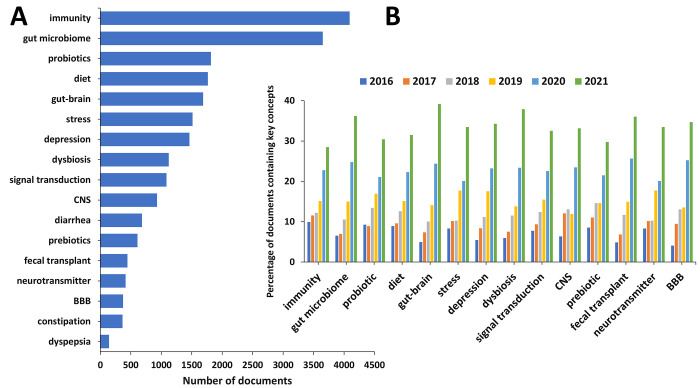
Key concepts in the scientific publications relevant to the gut microbiome research in mental and gut health. (A) Number of publications exploring key concepts related to gut microbiome research in mental and gut health. (B) Trends in key concepts presented in the articles related to gut microbiome research in mental and gut health during the years 2016–2021. Percentages are calculated with yearly publication numbers for each key concept, normalized by the total number of publications for the same concept in the same time period.

In order to better understand the advance in this research area, we examined the occurrence and trends of certain key concepts in the scientific publications relevant to the gut microbiome research in mental and gut health (Figure 5). With respect to the cumulative number of publications, “immunity” and “gut microbiome” appear as top concepts in the area (Figure 5A), thereby reflecting the rising interest in the relationship between the gut microbiome and systemic immune response pathways and the critical role the gut microbiome plays in training and development of the host’s innate and adaptive immune system. It is noteworthy that the concept concerning the gut–brain relationship exhibits the greatest growth rate in the past two years (Figure 5B), thereby characterizing it as the trendiest concept in the field.

## Gut-Microbiota-Participant Bacteria

The human gut microbiome, as mentioned above, is a complex mixture of microorganisms, including viruses, archaea, bacteria, yeasts, and fungi, interacting with each other and with their host in complex ways. These interactions at various times involve symbiosis, mutualism, antagonism, and even predation. Not only does the gut microbiome interact directly with the gastrointestinal (GI) tract, but it also interacts with the immune system that is present in the GI tract and with the neurological system through various signaling systems. Gastrointestinal signaling is mediated in part by microbial metabolites and is involved in regulating the gut–brain axis in the host.^85^ This section focuses on the bacterial microbiome by first discussing the techniques used to identify and study the gut microbiome. Then, we will discuss the human GI tract from an ecological viewpoint. The phyla commonly found in the gut microbiome will be defined and discussed. Finally, we will focus on probiotics and related compositions (prebiotics, postbiotics, synbiotics, and psychobiotics) and present recent examples of each class to illustrate the current state of the art.

### Techniques Used to Study the Gut Microbiome

Until recently, about 400 bacterial species were identified in the human microbiome using conventional culturing techniques.^86^ These techniques, by their nature, underestimate the actual number of species because, to culture a bacterium successfully, one needs to provide the correct nutrients, pH, and redox environment to enable growth. Conventional culture techniques favor fast-growing and nonfastidious species over those present in low concentration, which requires unusual culture conditions and/or complex nutritional requirements.^86^ Most isolation methods use selective agents, such as bile salts, to enrich the numbers of a desired bacterial type over others in the sample. The choice of the proper selective agent then becomes important. Some gut bacteria depend on other microorganisms in the gut to provide the nutrients they require for growth “cross-feeding.” Devising culture media for these can be a hit-or-miss proposition. Some bacteria will only grow in niches that have a narrow range of pH and/or redox potential, which may be difficult to maintain in vitro. Although there have been methodological advances in anaerobic culturing techniques, they tend to be tedious, time-consuming, and require specialized equipment.^31^ The successful culture of strict anaerobic bacteria requires training, experience, and careful planning. Additionally, some bacteria might be alive but unculturable. Intercellular adherence may reduce the number of organisms that can give rise to colonies.^87^

The development of culture-independent metagenomic approaches, such as 16S RNA gene sequencing and high-throughput sequencing, have been an enabling factor in the study of the human gut microbiome. This topic was reviewed in depth by Sankar et al. in 2015.^86^ The 16S rRNA gene exhibits several advantages, including its distribution in all bacterial species, its absence in eukaryotes, its stability over time, and its size (∼1500 bp), that make it suitable for bioinformatic analyses. High-throughput sequencing methods have given unprecedented access to the analysis of the microbial diversity of complex microbiotas, particularly through metagenomic approaches. Two strategies used are high-throughput sequencing of pooled PCR-amplified 16S rRNA and shotgun sequencing of all DNA fragments present, which enables identification of the microorganisms present and their metabolic genes. With these molecular techniques, it is now estimated that the human GI microbiota comprises more than 2000 species using modern molecular methods.^86,88^

Recent impressive advances in next generation sequencing technologies, along with the progress and innovations of metagenomics, metabolomics, multiomics, bioinformatics, and artificial intelligence tools, have provided prospects to better characterize the microbial populations and their functions and help in better correlation prediction.

### Gastrointestinal Tract

**Figure 6:**
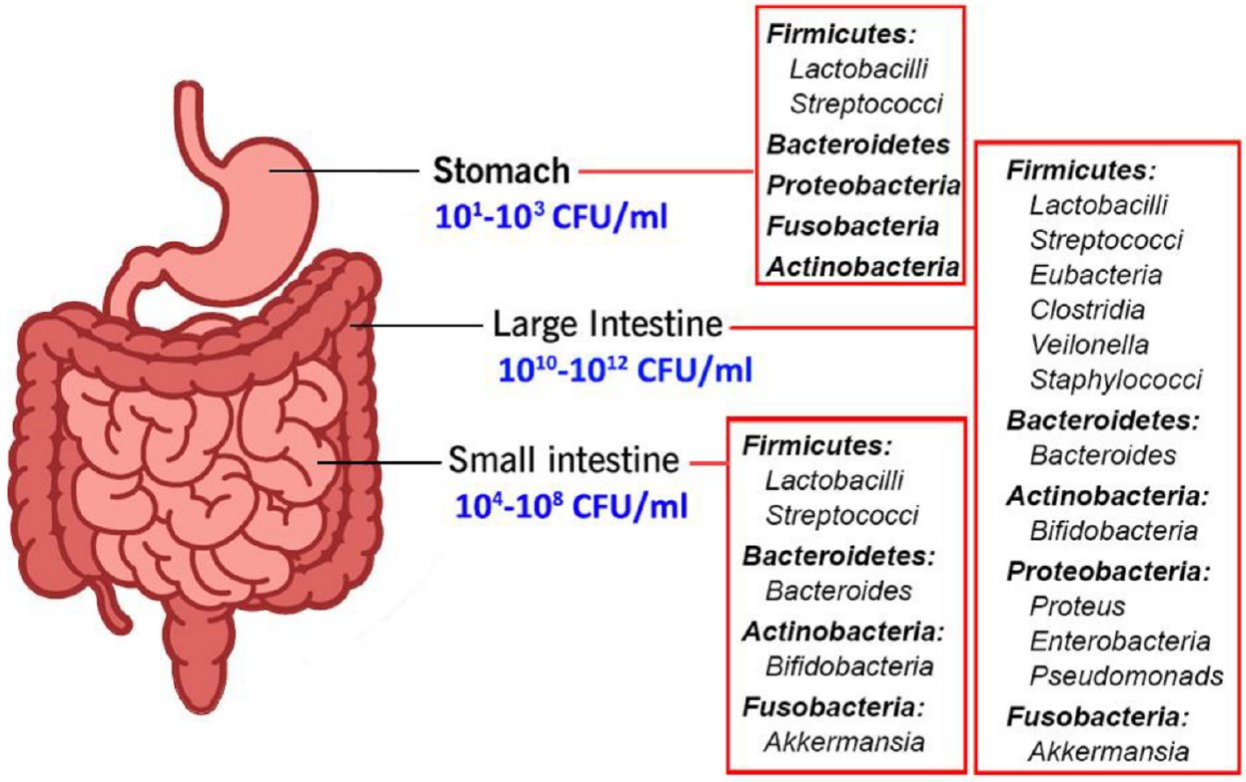
Gut-microbiota-participant bacteria.

The gut microbiome varies according to the GI anatomy, which varies in terms of physiology, pH and O_2_ tension, flow rates (rapid from the mouth to the cecum, slower afterward), substrate availability, and host secretions.^89^Figure 6 presents a representation of the GI tract with some of the bacterial taxa present. The GI tract consists of the stomach, duodenum, jejunum, ilium, cecum, and colon, with each environment ascending in pH and growing progressively more anaerobic from stomach to colon. Each section of the GI tract presents a unique ecological niche that exerts selective pressure on the microbiome. It is an open system with nutrients entering the system intermittently and wastes also leaving intermittently. The microbiome is affected by many factors, including diet, medications (especially antibiotics), ethnicity, age, and general health.^82,83^

The stomach is an extreme habitat because it is highly acidic (pH = ∼1.5). Once, it was considered sterile because of its acidity until the discovery of *Helicobacter pylori* in this hostile environment in 1982. The microbial population in the gastric environment is low and in the range of 10^1^–10^3^ cells/mL.^87^ Investigations since this discovery have revealed that the gastric fluid is predominated by the members of Firmicutes, Bacteroidetes, and Actinobacteria.^87^ The gastric mucosa was found to have a rich diversity with bacterial members belonging to Firmicutes, Bacteroidetes, Proteobacteria, Fusobacteria, and Actinobacteria. In healthy human stomach, the genera *Streptococcus*, *Prevotella*, *Veillonella*, *Rothia*, and *Haemophilus* were found to be predominant; however, the composition of the gastric microbiota is dynamic and affected by such factors as diet, drugs, and diseases.^90^

The small intestine comprises the duodenum, jejunum, and ileum. The duodenum has a pH of 5–6.8. Bacterial numbers are 10^3^–10^4^ cells/mL where Firmicutes predominate.^91^ The jejunum and ileum have a higher pH (6–8) with a 10^4^–10^8^ cell/mL density that comprises strict to facultative anaerobic Gram-positive and Gram-negative bacteria. The small intestine is lined with simple columnar epithelial tissue, which is covered by a mucus layer, and has a large surface area because of the villi and microvilli. When food enters the duodenum, the pH and bacterial load are low. These small intestinal mucosae are associated with members of phyla Bacteroidetes and Firmicutes. Food is blended with bile, bicarbonate, and digestive enzymes in the duodenum, and when the intestinal contents reach the large intestine, the food blend has been converted to a neutral to alkaline pH. The small intestine provides a more challenging environment for microbial colonizers given the short transit times (3–5 h) and the high bile concentrations.^83,91^

The large intestine consists of the cecum and colon and is characterized by slow flow rates and pH varying from 6 to 7.8. It harbors by far the largest microbial community. The large intestine is strictly anaerobic, and the cell density reaches 10^12^ cells/mL. The large intestine is home to the most complex bacterial diversity in the GI tract because of several factors, such as its larger volume, moderate or less acidic pH, low concentration of bile salts, and the longer retention time caused by slower peristalsis. Five major phyla—Firmicutes, Bacteroidetes, Actinobacteria, Verrucomicrobia, and Proteobacteria—covering a wide range of bacterial genera—*Clostridium*, *Fusobacterium*, *Bacteroidetes*, *Actinomyces*, and *Propionibacterium*—are associated with the large intestine. Other Gram-positive cocci—*Micrococci*, *Peptococci*, *Peptostreptococci*, and *Ruminococci*—have been also reported to play crucial roles in the large intestine. Food that has not been degraded in the upper GI tract reaches the large intestines and supports the microbiota with nutrients and energy. The carbohydrates present are fermented to carbon dioxide, hydrogen, methane, and short-chain fatty acids (SCFA) (primarily acetate, propionate, and butyrate). Most of the SCFA produced in the large intestine are absorbed by the host and provide an energy source. The amount of energy derived from SCFA accounts for 6–9% of the total energy requirement.^82,88,91,92^

The microbiome composition of the intestinal lumen, known as mucosal and epithelial spaces of the GI tract, is highly diverse and comprises Verrucomicrobia, Fusobacteria, Asteroplasma, Cyanobacteria, Actinobacteria, *Lentisphaera*, Spirochaetes, Bacteroidetes, Proteobacteria, Bacilli, Clostridia, and Mollicutes. The predominating genera are *Escherichia*, *Klebsiella*, *Enterococcus*, *Bacteroides*, *Ruminococcus*, *Dorea*, *Clostridium*, *Coprococcus*, *Weisella*, and *Lactobacillus*. Other genera found include *Granulicatella*, *Streptococcus*, and *Veillonella*.^83,91^

### Types of Bacteria Found in the GI Tract

**Figure 7:**
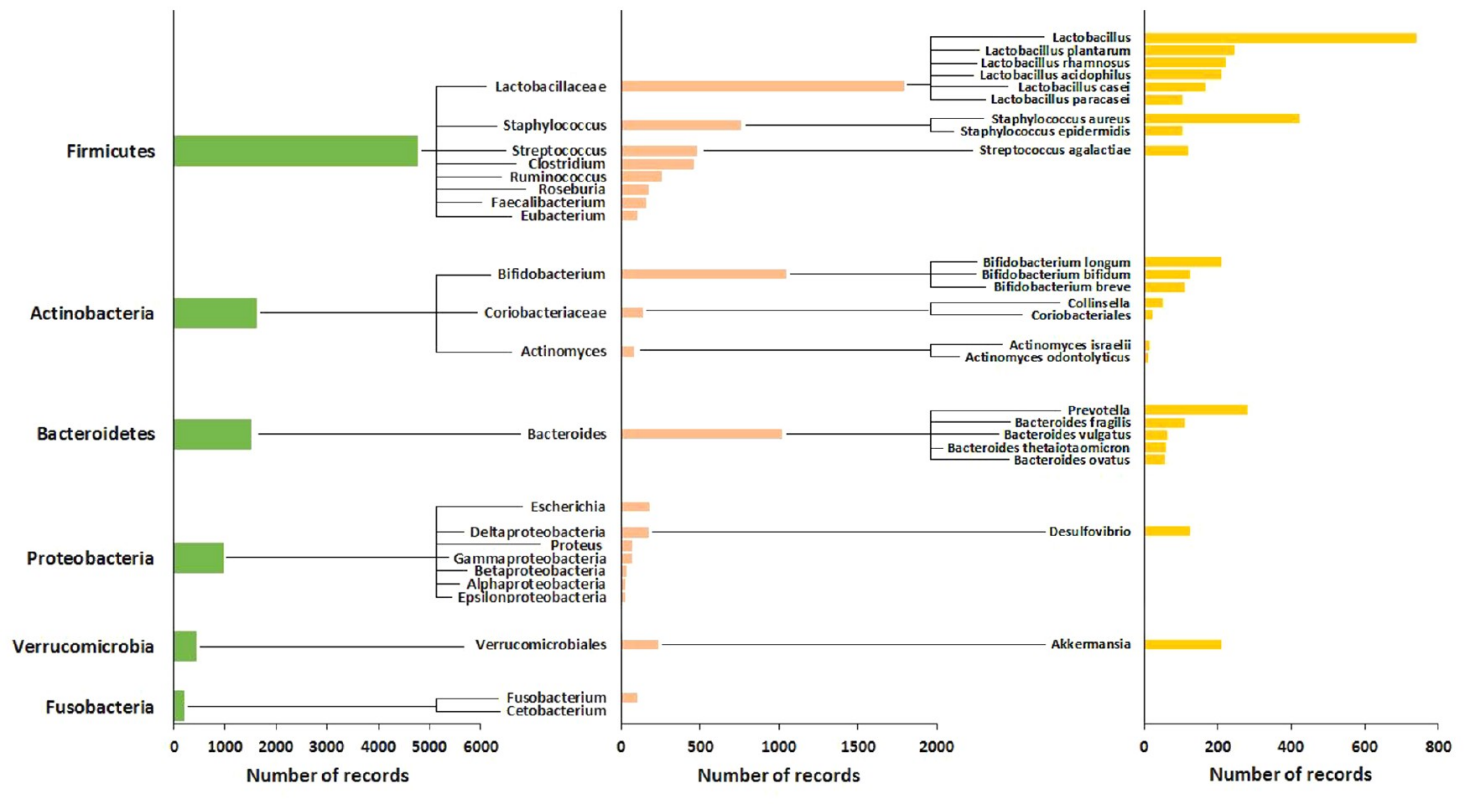
Representation (as number of records) of the gut bacteria phyla and species in the CAS Content Collection.

The four dominant phyla residents in the human gut are Firmicutes (which contains lactobacilli), Bacteroidetes, Actinobacteria (which contains *Bifidobacteria*), and Proteobacteria. Other phyla found in lower numbers are the Fusobacteria and Verrucobacteria. Figure 7 presents the significant phyla, families, and genera of gut bacteria in terms of the number of records that cite them in the CAS Content Collection. This presentation reflects the relative level of research interest in each of these taxonomic groups. Most bacteria belong to the genera *Bacteroides*, *Clostridium*, *Fusobacterium*, *Eubacterium*, *Ruminococcus*, *Peptococcus*, *Peptostreptococcus*, and *Bifidobacterium*. Other genera, such as *Escherichia* and *Lactobacillus*, are present to a much lesser extent. Twenty-three species from the genus *Bacteroides*, alone, constitute about 30% of all bacteria in the human gut.^93^

#### Bacteroidetes

Bacteroidetes are Gram-negative, nonspore-forming, anaerobic or aerobic, rod-shaped bacteria. *Bacteroides fragilis*, found in the human microbiome, is the type of species for this phylum. The majority of the Bacteroidetes species fall into three genera: *Prevotella* (bile-sensitive, moderately saccharolytic, with pigmented and nonpigmented species), *Porphyromonas* (bile-sensitive, pigmented, asaccharolytic species), and *Bacteroides* (bile-resistant, nonpigmented, saccharolytic species). Other genera in the phyla are *Alistipes*, *Anaerorhabdus*, *Dichelobacter*, *Fibrobacter*, *Megamonas*, *Mitsuokella*, *Rikenella*, *Sebaldella*, *Tannerella*, and *Tissierella*.^94^ Some members of the *Bacteroides* genus, although belonging to the normal gastrointestinal microbiota, can cause opportunistic infections if the integrity of the intestinal mucosal barrier is broken. These infections are usually polymicrobial, but *B. fragilis* and *B. thetaiotaomicron* are the most frequent species isolated. Some members of the genera *Porphyromonas*, *Prevotella*, and *Tannerella* are well-known pathogens of the oral cavity, where they can notably cause periodontal disease and dental caries.^95^ The ability of some members of the Bacteroidetes to degrade polysaccharides explains why they thrive in the GI tract.^96^

#### Firmicutes

The Firmicutes phylum comprises Gram-positive bacteria with low G + C DNA content and is composed of more than 200 different genera, such as *Lactobacillus*, *Bacillus*, *Clostridium*, *Enterococcus*, and *Ruminicoccus*.^82^ They can be found in a variety of places, including soil, water, skin, and the GI tract. The phylum includes aerobes, anaerobes, spore-forming, saprophytic, and pathogenic bacteria. Notable among the latter are *Clostridium difficile* and *Listeria monocytogenes*. Firmicutes, such as *Clostridium botulinum*, *Clostridium tetani*, *Clostridium perfringens*, and *Staphylococcus aureus*, can produce proteinaceous toxins. Other important genera are *Listeria*, *Paenibacillus*, *Staphylococcus*, *Streptococcus*, *Pediococcus*, and *Leuconostoc*.^97^ Some members of Firmicutes are involved in bile acid metabolism in the gut. Accumulating evidence suggests that bile acids play pivotal roles in gut inflammation and the development of intestinal bowel disease (IBD). Patients with IBD exhibit decreased microbial diversity and abnormal microbial composition marked by the depletion of phylum Firmicutes.^98^

#### Actinobacteria

The Actinobacteria are Gram-positive bacteria with high G + C DNA content and constitute one of the largest bacterial phyla. They are ubiquitously distributed in both aquatic and terrestrial ecosystems. Many Actinobacteria have a mycelial lifestyle and undergo complex morphological differentiation. They also have an extensive secondary metabolism and produce about two-thirds of all naturally derived antibiotics in current clinical use, as well as many anticancer, anthelmintic, and antifungal compounds. The phylum includes pathogens (species of *Corynebacterium*, *Mycobacterium*, *Nocardia*, and *Propionibacterium*), soil inhabitants (*Micromonospora* and *Streptomyces* species), plant commensals (*Frankia* spp.), and GI commensals (*Bifidobacterium* spp.)^99^ The *Bifidobacteria* are among the first microbial colonizers of the intestines of newborns and play key roles in the development of their physiology, including maturation of the immune system and use of dietary components. Some *Bifidobacterium* strains are considered probiotic microorganisms because of their beneficial effects, and they have been included as bioactive ingredients in functional foods, mainly dairy products, as well as in food supplements and pharma products, alone or together with other microbes or microbial substrates.^100^

#### Proteobacteria

The name Proteobacteria was first proposed by Stackebrandt et al. in 1988.^101^ The name was derived from Proteus the ancient Greek god of the sea capable of assuming different shapes, which reflected the high heterogeneity displayed by the bacteria belonging to this phylum. A common trait of Proteobacteria is Gram-negative staining, which indicates the presence of lipopolysaccharide in the outer membrane. On the basis of phylogenetic analysis of the 16S rRNA gene, the Proteobacteria phylum is divided into six classes (previously regarded as subclasses of the phylum): Alphaproteobacteria, Betaproteobacteria, Gammaproteobacteria, Deltaproteobacteria, Epsilonproteobacteria, and Zetaproteobacteria. Considering that the classes division is based on molecular relatedness, it is not surprising that no specific morphological or physiological trait characterizes members within each class.^102^ Notable genera in the Proteobacteria are *Escherichia*, *Salmonella*, *Shigella*, *Desulfovibrio*, and *Helicobacter*. Included in the phyla is the Enterobacteriaceae family, which contains several enteropathogenic bacteria, including *Shigella flexneri*, *Salmonella typhi*, and *Escherichia coli*. Other enteric pathogens in this phylum are *Vibrio cholerae* and *Helicobacter pylori*.

#### Verrucomicrobia

The phylum Verrucomicrobia, like the Proteobacteria, is defined by as a distinct phylogenetic lineage, as determined by 16S rRNA gene sequences. The phylum has been recognized as separate since 1995 but currently counts only a few cultivated microorganisms as members. Verrucomicrobia is a divergent phylum that includes members of the microbial communities of soil and fresh and marine waters. Some extremely acidophilic members from hot springs have been found to oxidize methane.^103,104^*Akkermansia muciniphila* is a mucus-degrading member of the Verrucomicrobia found in the human GI tract. *A. muciniphila* represents from 1 to 4% of the bacterial population in the colon.^105^*A. muciniphila* prefers to colonize in the intestinal mucus layer and specifically degrades mucins to produce short-chain fatty acids, thereby providing energy for the host and promoting colonization of the bacterium itself. The degradation of mucins prompts the host to compensate with the production of more mucins, thereby maintaining the dynamics of these proteins.^106^

#### Fusobacteria

The phylum Fusobacteria is made up of Gram-negative, nonmotile, facultative aerobic to obligately anaerobic, fermentative, rod-shaped bacteria, which have generally fusiform (spindle-shaped) morphology. Fusobacteria have been known for more than 100 years, but recently, phylogenetic studies have shown that they should be grouped into a distinct phylum. The bacteria from this phylum are commonly associated with the mucous membrane of humans and animals. They are also commonly present in the human and animal GI tract, particularly in the jejunum, the ileum, and the colon.^83,107^

### Gut Microbiome–Disease Correlations

**Figure 8:**
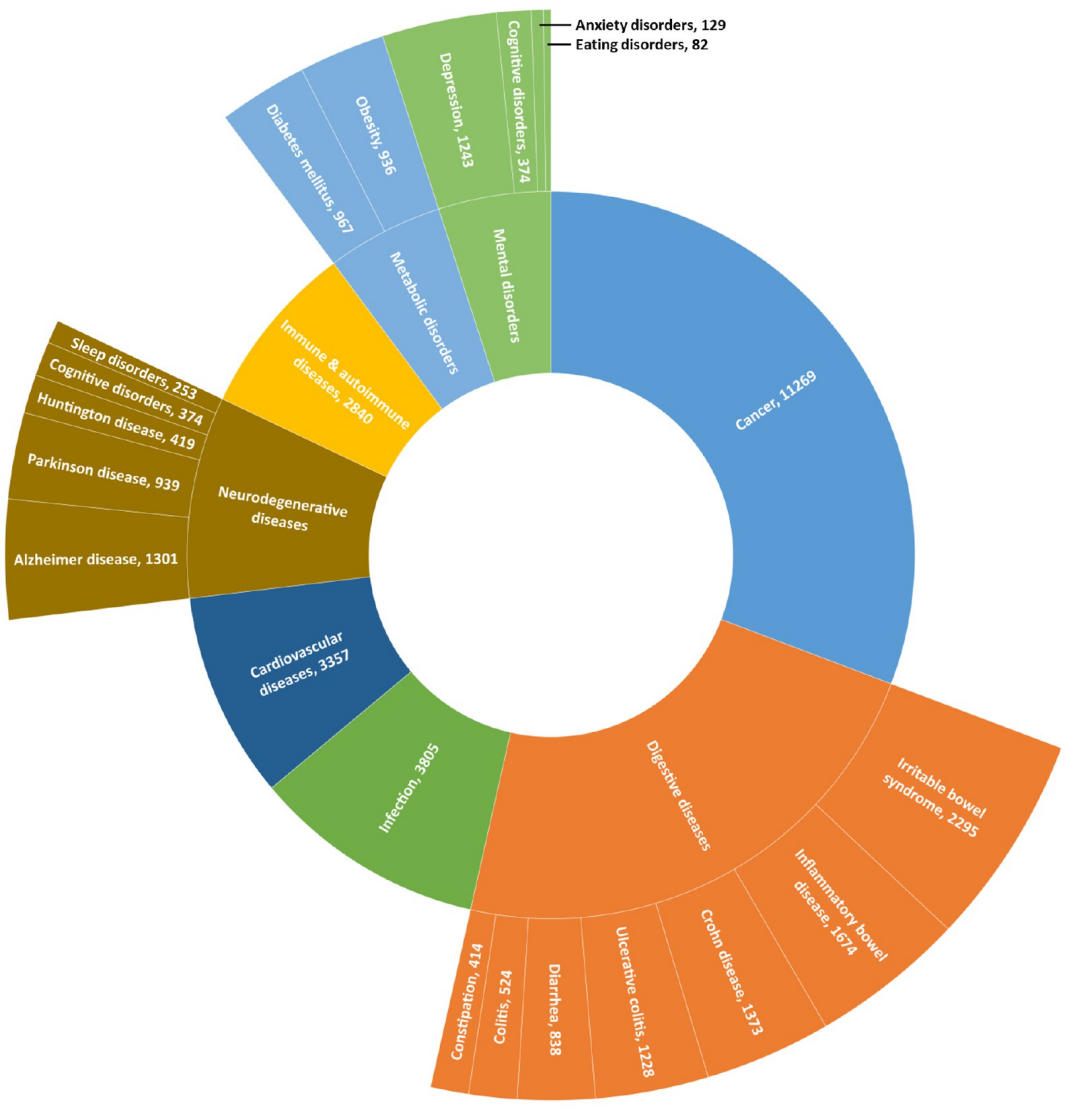
Distribution of the publications in the CAS Content Collection related to gut microbiome-associated diseases.

**Figure 9:**
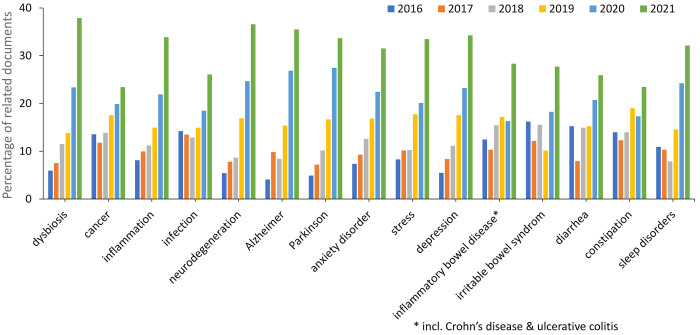
Trends in the number of publications concerning gut microbiome-related diseases during the years 2016–2021. Percentages are calculated with yearly publication numbers for each disease, normalized by the total number of publications for the same disease in the same time period.

The human microbiome has been recognized as an essential factor for human health.^108−110^ Specifically, gut microbes contribute directly and/or indirectly to important physiological activities, including immunomodulation and the regulation of various neurotransmitters, hormones, and metabolites. Dysbiosis is a state characterized by distinct alterations in the microbiome that result in an imbalance in the microbiota, modifications in their functional composition and metabolic performance, or a change in their allocation. The impact of the microbiome on human physiology and pathology is so extensive that the microbiome has been considered as an essential organ of the human body.^111−113^ A search in the CAS Content Collection identified a large collection of studies reporting correlations between gut microbiota and a wide range of diseases, including mental, metabolic, and digestive system disorders; cardiovascular and neurodegenerative diseases; various cancers; and immune and autoimmune diseases (Figure 8). Trends in the number of publications related to various diseases in the recent years (2016–2021) are depicted in Figure 9. The number of documents related to dysbiosis, in general, exhibits the greatest growth rate, thereby characterizing the dominant fundamental approach of the recent studies in the field.

#### Digestive System Diseases and Disorders

Alterations to the gut microbiota composition have been associated with various digestive system disorders and diseases, specifically IBS; IBD, including Crohn’s disease and ulcerative colitis; diarrhea, and constipation (Figure 8).^114−116^

Irritable bowel syndrome is one of the most prevalent functional gastrointestinal disorders and is considered as the prototype of disorders of the gut–brain interaction. While alterations in gut–brain interactions have clearly been established in IBS, a causative role of the microbiome remains to be determined. Dysbiosis is one of the hallmarks in the miscommunication between gut and brain and could lead to IBS symptoms. The severity of IBS symptoms has been shown to be correlated with dysbiosis.^117,118^ In IBS cases, the reduction of microbiome diversity, gut barrier deficiency, gut–brain signaling disorders, and immune disorders are significantly related to the abnormal function of the GI tract.^119^ Modifications in the composition of the normal microbiota and perturbed colonic fermentation in IBS patients are supposed to play a role in the development of IBS, with a considerable, nearly 2-fold, increase in the ratio of Firmicutes to Bacteroidetes.^120^ Recent studies have reported well-defined distinction between gut microbiota composition in patients with IBS compared with healthy controls. IBS was typified by enhanced quantities of Firmicutes and specifically in *Ruminococcus*, *Clostridium*, and *Dorea*, along with a distinct decrease of beneficial microbes, such as *Bifidobacterium* and *Faecalibacterium* spp.^121^ Moreover, a decrease in probiotic species and an increase in pathogenic species have been reported in patients with IBS, including Proteobacteria, Enterobacteriaceae, Lactobacillaceae, and *Bacteroides* (Bacteroidetes).^122^ Fecal transplantation from super donors and microbiome modulation either by pro- or prebiotics have shown beneficial effect in reducing IBS symptoms and improving patients’ quality of life.^119,123,124^ To date, the guidelines on the treatment of IBS with probiotics remain controversial. The British Society of Gastroenterology guidelines^125^ on the management of IBS, which were updated in 2021, reported that probiotics may be an effective treatment for improving global symptoms and abdominal pain in patients with IBS, which was consistent with the recommendations of the Canadian Association of Gastroenterology^126^ and the Japanese Society of Gastroenterology.^127^ In contrast, the guidelines from the American College of Gastroenterology^128^ suggest against the use of probiotics for the treatment of global IBS symptoms.^129^

Like IBS, inflammatory bowel disease-related dysbiosis is associated with a general decrease in richness, diversity, and stability of the microbiota.^130^ This decline in diversity is concomitant with a weakened immune response and setbacks with the cellular barrier functions that normally block bacterial entry from the gut lumen into gut tissue. These malfunctions trigger complications with antibacterial defense and consequent growth of pathogenic bacteria.^131^ IBD-related dysbiosis is specifically associated with a comprehensive decrease in the quantity and diversity of Firmicutes and an increase in Proteobacteria.^132^ The decrease in the numbers of Firmicutes is noteworthy since they produce essential short-chain fatty acids, such as acetic and butyric acids, that are known to exhibit anti-inflammatory properties.^133^ A common feature of the microbial dysbiosis among IBD patients, especially in Crohn’s disease, is the decreased abundance of Firmicutes bacteria belonging to two families that are important functional members of the human gut microbiota—Ruminococcaceae and Lachnospiraceae—to which most butyrate-producing bacteria in the human gut belong.^134,135^ Thus, depletion of these bacterial families in IBD is supposedly correlated to the detected disturbances, such as a lower butyrate-producing capacity of the IBD microbiota.^136^ Butyrate has a significant potential in IBD therapy because it serves as the colonocytes key energy source, enhances the epithelial barrier integrity, and inhibits inflammation. A probiotics treatment, including consumption of butyrate-producing bacteria to increase *in situ* butyrate production, may restore gut homeostasis.^137−139^ A recent study reported that an orally delivered cocktail of bacteriophages targeting an IBD-associated strain of the bacterium *Klebsiella pneumoniae* alleviated intestinal inflammation.^131^

A growing body of evidence shows that imbalance of the gut microbiota increases susceptibility to various pathogens and causes numerous diseases, including diarrhea.^140^ At present, the pathogens causing diarrhea are believed to be *Escherichia coli*, *Shigella*, *Salmonella*, *Campylobacter*, *Clostridium difficile*, and *Aeromonas*.^141,142^ It has been found that microbial intervention can regulate the composition of the intestinal flora to prevent and improve the occurrence of diarrhea.^143^ Probiotics containing nonpathogenic live bacteria preparations, such as *Lactobacillus*, Yeast, *Bifidobacterium*, *Enterococcus*, and *Bacillus*, have been demonstrated to treat pathogens-caused diarrhea by preserving or amending the balance of gut microbiota. The mechanisms of the beneficial effect are supposedly related to the inhibitory effect on the colonization of pathogenic bacteria by competing for nutrients and producing antibacterial compounds.^144^

Accumulating evidence suggests an association between functional constipation and abnormal gut microbiota, with the relationship between gut microbiota and gut transit being likely bidirectional.^145^ By controlling colonic motility, water content, secretion, and absorption, gut microbiota may promote the development of functional constipation through microbial metabolites, including bile acids, SCFAs, 5-hydroxytryptamine, and methane. Currently, there is no consensus on the gut microbial composition typical for functional constipation patients and the alteration trends of the various microbial classes compared with healthy controls.^146−150^ However, recent studies showed that changes in the mucosal and fecal microorganisms are linked to functional idiopathic constipation. Taxonomic profiling of intestinal microbiota in constipated adults showed a higher abundance of *Bacteroides* and other pathogenic microorganisms than in healthy volunteers.^151^ The increased richness and diversity of the gut microbiomes result in slow colonic transit. In addition, intestinal microbiota in constipated adults have genes involved in pathways that lead to methane, hydrogen, and glycerol production, which can explain the symptoms seen in patients with constipation.^151−153^ Microbial interventions including probiotics, prebiotics, and synbiotics, which bring about compositional and functional changes of the gut microbiota, have frequently shown beneficial effects on functional constipation that are in favor of the concept of the significant role of gut microbiota in functional constipation.^145^ This concept is supported also by the reports that many risk factors of functional constipation, including age, diet, obesity, and stress, have a considerable effect on the gut microbiota.^154,155^

#### Mental and Neurodegenerative Disorders

Gut microbiota have been reported to affect neurological functions along the so-called gut–brain axis (GBA).^156^ Gut microbiota communicates with the brain through three major routes: the neural route (vagus nerve, enteric nervous system), the immune route (cytokines), and the endocrine route [hypothalamus–pituitary–adrenal (HPA) axis, gut hormones]. Disturbances in any of these routes can result in mental disorders. Dysbiosis in common intestine microbial species of the phylum Firmicutes and Actinobacteria and the genera *Bacteroides* and *Bifidobacterium* are supposedly responsible for mental health disorders.^157^ Gut microbiota moderate the GBA via various ways, such as preserving gut permeability by controlling the integrity of tight junctions in gut epithelium and producing a large selection of metabolites that include neurotransmitters, SCFAs, and amino acids.

A plethora of research reports have indicated the significance of microbiota in the development of neurodegenerative diseases via a variety of microbial metabolites that transmit from the gut to the brain across the GBA.^158,159^ Changes in the levels of gut microbial metabolites have been reported to be associated with neurological conditions like Parkinson’s disease,^160^ anorexia nervosa,^161^ Alzheimer’s disease,^162^ autism spectrum disorders,^163^ and chronic stress and depression.^164^ It is not clear by now, however, whether these disruptions in mental health are the cause or a result of the changes in gut microbiota. Gut dysbiosis has been associated with increased gut permeability and inflammation and it may also cause enhanced levels of circulating gut microbiota metabolites, such as the neurotoxin β-*N*-methylamino-l-alanine and microbial amyloids.^165,166^ β-*N*-Methylamino-l-alanine is one of the gut cyanobacteria-produced neurotoxins causing neurodegeneration, cognitive impairment, and the accumulation of neurofibrillary tangles.^167,168^ The hypothesis that Parkinson’s disease starts in the gut and spreads to the brain^169^ is gaining increasing support, thereby showing that the disease is associated with widespread dysbiosis.^170^

Alterations in the microbiota composition in patients with Alzheimer’s disease compared with matched healthy controls included a reduction in richness and diversity of gut microbiota with decreased Firmicutes and *Bifidobacterium* and increased Bacteroidetes.^171^ Changes in Actinobacteria, *Ruminococcus*, Lachnospiraceae, and Selenomonadales (1686) have also been reported.^172^ Cognitively impaired patients exhibited alterations in Bacteroidetes, Firmicutes, Proteobacteria, and Verrucomicrobia compared with age-matched cognitively intact individuals.^173^

Therapeutic interventions including the administration of pre- and probiotics (psychobiotics) to manage mental disorders and/or their symptoms have been undertaken.^174,175^ These inventions have included probiotic combinations of lactobacilli and *Bifidobacteria*, which has resulted in a significant drop in psychological distress,^176^ enhanced cognition and communication among patients with Alzheimer’s disease^177^ and autism spectrum disorders,^178^ and recovering symptoms among patients with Parkinson’s disease.^179^ On the basis of the promising results of psychobiotics on controlling or modulating the GBA, additional clinical trials are currently being undertaken to identify bacterial strains as promising candidates for the treatment of mental disorders.

Humans are adapted to a circadian rhythm of 24 h associated with the light/dark cycle on earth. The central circadian clock is located in the hypothalamus, which synchronizes information on environmental light and dark signals to peripheral tissues to keep the body functioning in the same rhythm.^180^ A disruption of circadian rhythms is associated with various diseases, including neurodegenerative diseases, sleep, and psychiatric disorders.^181,182^ Recent studies have reported that gut microbiota are able to control or be controlled by the circadian clock. The mechanisms of such a relationship requires small molecule gut microbiota metabolites, such as bile acids and SCFAs, to act as intermediaries.^180^ Thus, the levels of butyrate and propionate show obvious daily oscillations. Moreover, these oscillations are lost under high-fat diets.^183^ The impacts of gut microbiota metabolites on circadian rhythm are extensive and are connected to other functions of gut microbiota metabolites, such as energy metabolism and immunity. These interconnections between different physiological functions via the link of gut microbiota metabolites are essential for understanding the functions of gut microbiota metabolites and the general role of gut microbes in human health and disease.

#### Metabolic Disorders

Systemic metabolic diseases that are believed to be strongly affected by gut microbiota status include obesity and diabetes.^184^ Gut microbial composition is strongly affected by dietary routines. As a result of a high-fat diet, the intestinal microbiome is modified with rising amounts of Firmicutes and Proteobacteria and reduced levels of Bacteroidetes. The Firmicutes/*Bacteroides* ratio has been correlated to body weight, which means it is larger for obese people.^185^*Clostridium difficile* infections can also trigger obesity. Generally, obesity is affected by the inflammatory status induced by gut bacteria or their metabolites, which regulate the GBA.^108,185^

Diabetes is another metabolic disease that is strongly associated with the gut microbiome. Studies have reported an increased quantity of *Villanella*, *Clostridium*, and *Bacteroides* and a decreased quantity of *Lactobacillus*, *Eubacterium rectale*, *Blautia coccoides*, and *Bifidobacterium* in children with type 1 diabetes. Besides, negative correlation has been reported between plasma glucose level and *Bifidobacterium*, *Lactobacillus* spp., and Firmicutes and Bacteroidetes spp., while there has been positive correlation between *Clostridium* and plasma glucose level. The ratios of Bacteroidetes to Firmicutes were reported to exhibit a positive connection with plasma glucose levels. The *Lactobacillus* genus was also in lower quantity in type 2 diabetes patients, and *Bifidobacterium* was in higher quantity compared with control groups.^108,186^ Risks for the development of type 2 diabetes have been correlated to the composition of gut microbiota, as well. The alterations in the gut microbiota of individuals with type 2 diabetes have been small compared with the control group, yet a consistent decline in the metabolically beneficial butyrate-producing bacteria was reported.^186^ Overall, type 2 diabetes was associated with a reduced quantity of SCFAs-producing bacteria, in particular butyric acid, which has been related to insulin sensitivity.^187,188^ The relation between SCFAs and insulin sensitivity stems from the capacity of SCFAs to stimulate the secretion of GLP-1 by intestinal L-cells via G protein receptors, which has a significant impact on insulin release.^189^

Close relations between the metabolic and immune systems are now largely supported, and intestinal microbiota is being progressively identified as an important factor connecting genes, environment, and the immune system.^190^

#### COVID-19

Recently, correlation has been reported between gut microbiota composition and levels of cytokines and inflammatory markers in patients with COVID-19.^191,192^ It is suggested that the gut microbiome is involved in the magnitude of COVID-19 symptoms’ severity via modulation of the host’s immune responses. Moreover, gut microbiota dysbiosis could contribute to persistent symptoms even after disease resolution, thereby emphasizing a need to understand how gut microorganisms are involved in inflammation and COVID-19.A recent study demonstrated that SARS-CoV-2 infection indeed disrupts the gut microbiome.^193^ This boosts secondary bacterial infections both by facilitating pathogenic bacteria to colonize the gut and by modifying gut lining to allow these bacteria to spread from the gut to the bloodstream of COVID-19 patients. These results confirm the direct role of gut microbiome dysbiosis in facilitating grave secondary infections upon COVID-19 malady.^193^

The alterations to the gut microbiota composition related to digestive system diseases, mental health, and metabolic disorders are summarized in Table 1.

**Table 1:** Gut Dysbiosis in Digestive System Diseases, Mental Health, and Metabolic Disorders

diseases	↓ decreasing bacteria	↑ increasing bacteria
digestive system diseases		
irritable bowel syndrome^194−199^	*Bifidobacterium*	*Ruminococcus*
	*Faecalibacterium prausnitzii*	*Dorea*
	*Bacteroides*	Enterobacteriaceae
		Lactobacillaceae
		*Bacteroides*
		Firmicutes/Bacteroidetes ratio
IBD: Crohn’s disease^200,201^	*Bacteroides*	
	*Faecalibacterium prausnitzii*	
	*Bifidobacterium adolescentis*	
IBD: ulcerative colitis^139,200^	*Bifidobacteria*	
	*Roseburia hominis*	
	*Faecalibacterium prausnitzii*	
	Lachnospiraceae	
	Ruminococcaceae	
mental health disorders		
anxiety disorder^202,203^	Bacteriodetes	Bacteroidaceae
	*Ruminococcus gnavus*	Enterobacteriaceae
	*Fusobacterium*	Burkholderiaceae
post-traumatic stress disorder^204^	Actinobacteria	
	Lentisphaerae	
	Verrucomicrobia	
depression^205−207^	*Prevotella*	*Eggerthella*
	*Dialister*	*Holdemania*
		*Turicibacter*
		*Paraprevotella*
dementia^172^	Actinobacteria	*Escherichia*
	*Bacteroides*	*Blautia*
		*Bifidobacterium*
		*Streptococcus*
		*Lactobacillus*
		*Dorea*
metabolic disorders		
diabetes type 1^208,209^	*Lactobacillus*	*Clostridium*
	*Bifidobacterium*	*Bacteroides*
	*Blautia coccoides*	*Veillonella*
	*Eubacterium rectale*	Actinobacteria
	*Prevotella*	Proteobacteria
	*Akkermansia*	*Lactococcus*
	Firmicutes	
diabetes type 2^199,209,210^	Firmicutes	Betaproteobacteria
	Clostridia	
	*Lactobacillus*	Bacteroidetes/Firmicutes ratio
	*Akkermansia muciniphilia*	
	*Roseburia*	
obesity^199,211^	Bacteroidetes	Enterobacteria
	*Methanobrevibacter smithii*	*Ruminococcus gnavus*
	*Ruminococcus flavefaciens*	Actinobacteria
	*Bifidobacterium*	Prevotellaceae

#### Gut Bacteria–Disease Correlations

**Figure 10:**
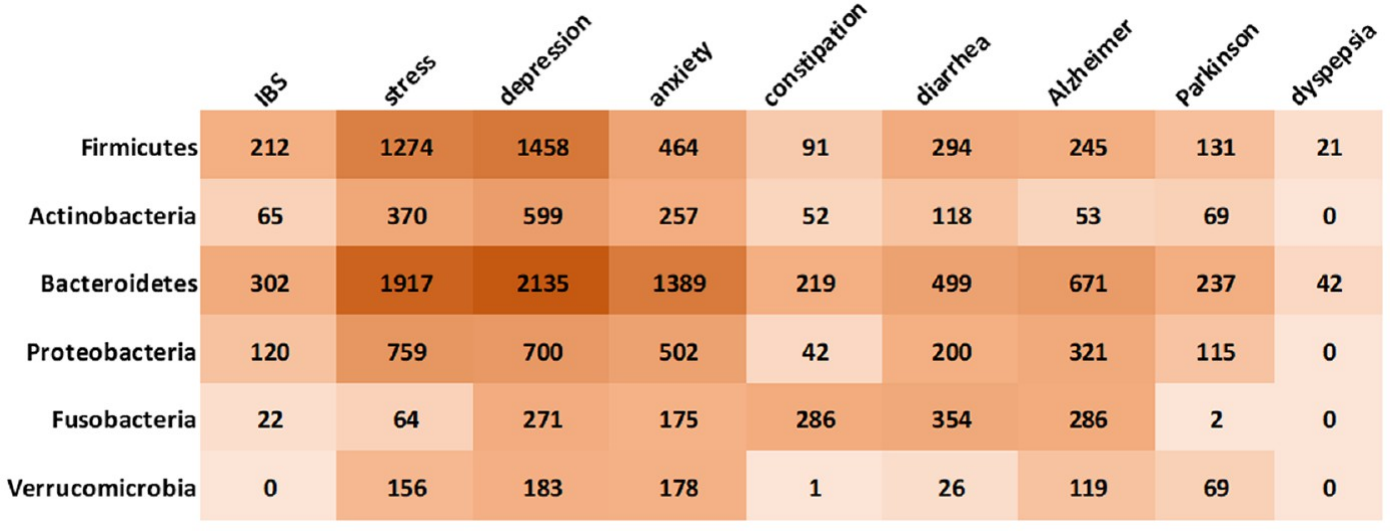
Correlation between major classes of gut bacteria with mental and gastrointestinal disorders, as reflected in the number of associated records in the CAS Content Collection.

In an effort to get better insight into the gut microbiota impact on well-being, we explored the correlations between the major classes of gut bacteria and certain mental and gastrointestinal disorders, as reflected in the number of records in the CAS Content Collection (Figure 10).

As seen from Figure 10, Bacteroidetes are the most studied class of gut bacteria with relation to gastrointestinal and mental health, specifically with relation to stress, depression, and anxiety. Bacteroidetes are known to have a very broad metabolic potential and are regarded as one of the most stable parts of gastrointestinal microflora that exhibits remarkable nutritional flexibility and an ability to respond to stress.^212^ However, the exact mechanisms underlying any possible relationship between Bacteroidetes and mental and gastrointestinal health remains unclear, and further research is needed to fully understand this complex relationship.

Firmicutes are the second extensively studied class of gut bacteria with relation to gastrointestinal and mental health, especially in relation to stress and depression (Figure 10). Many members of the Firmicutes phylum, such as *Lactobacillus*, are probiotic. The relationship between Firmicutes and gastrointestinal disorders may be mediated by a number of different factors, such as the production of SCFAs by Firmicutes, which are an important energy source for the gut epithelium and have anti-inflammatory effects. In addition, some Firmicutes bacteria are involved in the fermentation of complex carbohydrates and the production of beneficial metabolites, such as butyrate, which has been shown to have protective effects against colorectal cancer.^213^ Like with Bacteroidetes, the exact relationship between Firmicutes and mental and gastrointestinal disorders is still being studied and is not fully understood.

The interest in Fusobacteria with respect to the gastrointestinal and mental health is mainly related to diarrhea and constipation (Figure 10). Recent evidence is emerging that this bacterium may be related to human colon cancer.^214^

It is noteworthy that recent meta-analysis has reinforced the genetic correlations between Alzheimer’s disease and the gut microbiome genera.^215^ For example, genus Actinobacterium *Collinsella* was confirmed to be associated with Alzheimer’s disease, as well as rheumatoid arthritis, atherosclerosis, and type 2 diabetes.^215^ It is also worth reiterating that the gut microbiota is a complex and diverse community of microorganisms, and changes in any one particular phylum are unlikely to fully explain the development of any particular disorder or condition. Rather, the gut microbiota as a whole are likely to play a role in shaping our physical and mental health, and further research is needed to fully understand the complex relationships between the gut microbiota and overall well-being.

#### Therapeutic Strategies for the Treatment of Mental and Gastrointestinal Disorders

Imbalances within the gut microbiome–brain axis have been linked to a range of mental and gastrointestinal disorders. Assorted therapeutic interventions aimed at modulating the gut microbiome or the gut–brain axis may be effective in improving outcomes for these conditions.

##### Dietary Interventions

A high-fiber diet can increase the production of SCFAs, which can help to maintain gut barrier function and reduce inflammation. SCFAs can also promote the growth of beneficial gut bacteria, which can outcompete pathogenic bacteria and reduce inflammation.^216^

A diet low in fermentable oligosaccharides, disaccharides, monosaccharides, and polyols (FODMAP) can reduce symptoms in individuals with irritable bowel syndrome by reducing the fermentation of certain carbohydrates in the gut, which can cause symptoms such as bloating and abdominal pain. However, the low-FODMAP diet can also reduce the diversity of gut bacteria and may have negative long-term effects on gut health.^217,218^

##### Probiotics and Prebiotics

Probiotics can improve gut barrier function by enhancing the production of mucus and tight junction proteins, which can prevent the entry of harmful molecules and pathogens into the bloodstream. They can also reduce the production of proinflammatory cytokines and modulate the activity of immune cells in the gut, thereby promoting an anti-inflammatory response.^219,220^

Prebiotics can increase the production of metabolites, such as SCFAs, which are important energy sources for gut cells and can help to maintain gut barrier integrity. SCFAs can also activate G protein-coupled receptors on immune cells, thereby leading to the production of anti-inflammatory cytokines and the suppression of proinflammatory cytokines.^221,222^

##### Antibiotics

Antibiotics can kill harmful bacteria in the gut, which reduces inflammation and restores gut barrier function. However, antibiotics can also have negative effects on the gut microbiome, such as by reducing the diversity of gut bacteria and promoting the growth of antibiotic-resistant bacteria. Antibiotics should be used judiciously and only when necessary.^223,224^

##### Fecal Microbiota Transplantation (FMT)

FMT can restore the composition and function of the gut microbiome, which can reduce inflammation and improve gut–brain communication. FMT has been shown to be effective in treating recurrent *Clostridium difficile* infection and is being investigated for other conditions, such as IBD and autism.^225−227^

##### Psychotherapeutic Interventions

Psychotherapeutic interventions can reduce stress and improve gut–brain communication. Stress can disrupt gut microbiome composition and function, which leads to inflammation and the development of diseases related to the gut microbiome–brain axis. The reduction of stress can be an effective way to improve gut health and reduce symptoms in these conditions.^228−230^

##### Pharmacological Interventions

Drugs that target the gut–brain axis can modulate gut microbiome composition and function and reduce inflammation. For example, certain drugs that target the serotonin system, such as selective serotonin reuptake inhibitors (SSRIs), can modulate gut–brain communication and have been shown to be effective in treating conditions, such as depression and anxiety.^231,232^

**Figure 11:**
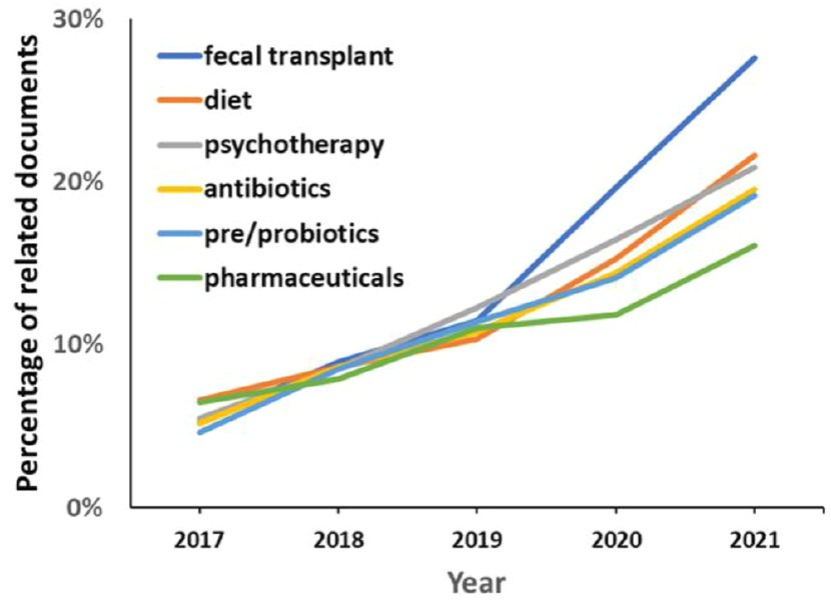
Trends in the therapeutic strategies applied for the treatment of mental and gastrointestinal disorders, as presented in the documents related to gut microbiome research during the years 2017–2021. Percentages are calculated with yearly publication numbers for each type of therapeutic intervention normalized by the total number of publications for the same intervention in the same time period.

Figure 11 demonstrates the annual growth in the number of documents in the CAS Content Collection related to various therapeutic interventions applied for the treatment of mental and gastrointestinal disorders. Fecal transplant as a new therapeutic strategy exhibits the fastest growth rate recently.

The various strategies to treating diseases related to the gut microbiome–brain axis can work through multiple mechanisms, including modulating gut microbiome composition and function, reducing inflammation, and improving gut–brain communication.^233^ However, the underlying mechanisms are complex and require further research to fully understand.

## Gut Microbiota Metabolites

It is estimated that the human gut microbiome contains more than 22 million microbial genes,^234^ which exceeds the ∼22,000 genes present in the entire human genome.^235^ These genes enable the gut microbiota in the host to synthesize a myriad of enzymes with versatile capabilities to ferment and degrade a variety of compounds that humans do not have the genetic machinery to metabolize. As a result, the gut microbiota can generate a battery of metabolites with a wide spectrum of bioactivities. The gut microbiota-derived metabolites can be broadly divided into three types according to their origination: (1) metabolites that are produced by gut microbiota directly from diets; (2) metabolites that are generated by the host and modified by gut microbiota; (3) metabolites that are produced de novo.^236^ A selection of important gut microbiota metabolites related to gut–brain communication is shown in Table 2.

**Table 2:**
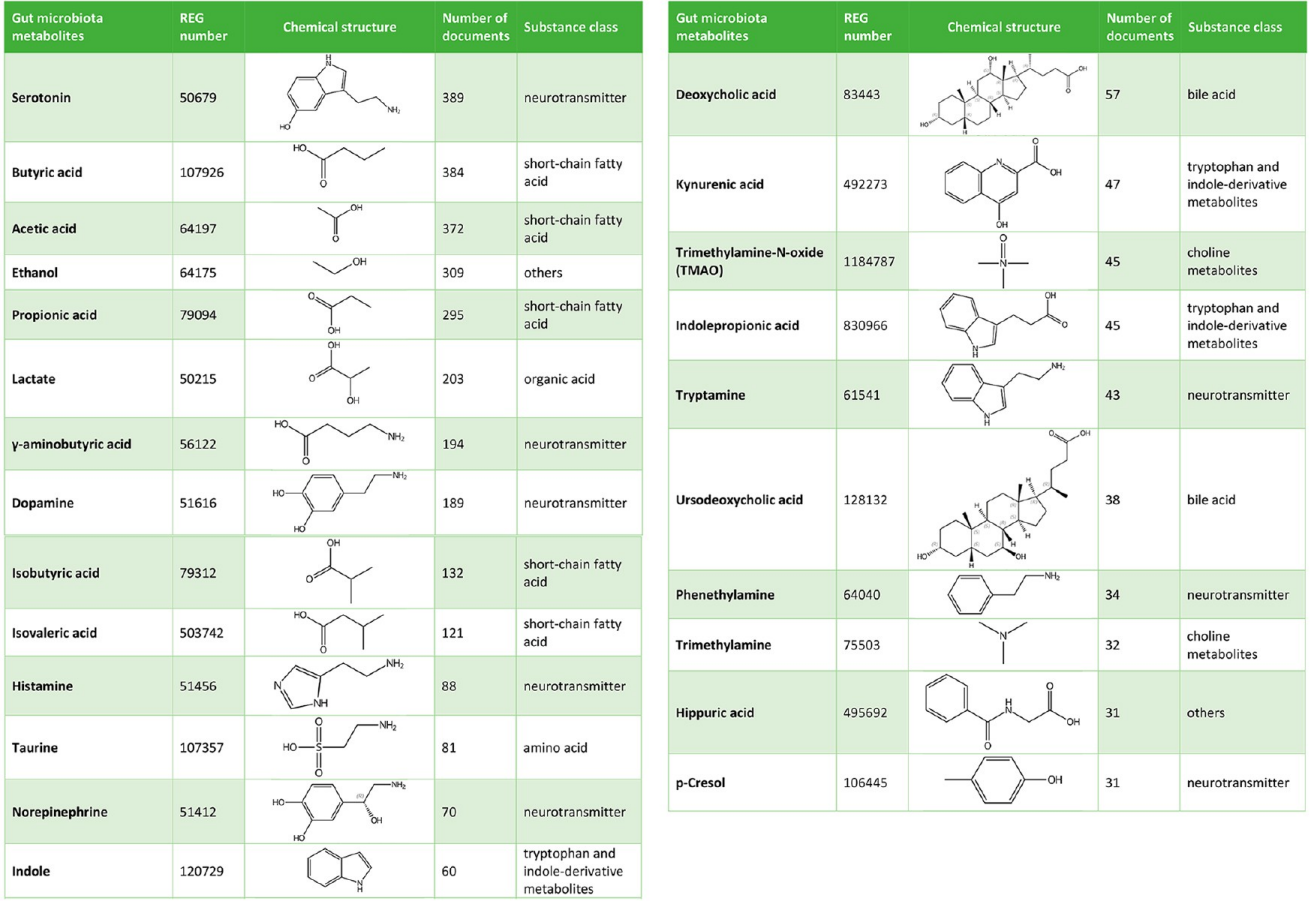
Exemplary Gut Microbiota Metabolites, as Represented in the CAS Content Collection

### Gut Microbiota Metabolites with Impact on Brain Function

The gut microbiota provides essential signaling metabolites that are vital for the host’s physiology. While in healthy individuals, gut microbiota metabolites are effective in maintaining the important functions of hosts, perturbations in the production of these metabolites can initiate various diseases, such as digestive system diseases, neurodegenerative and metabolic disorders, and cancer.^236^

Research has shown that gut microbiota is crucial for normal brain development and function.^237,238^ For example, administration of 4-ethylphenylsulfate (4-EPS), a tyrosine derivative, to mice at 3–6 weeks postnatal induced anxiety-like behavior. The biosynthetic pathway analysis and mechanisms behind the detrimental effects of 4-EPS showed that 4-EPS interfered with oligodendrocyte maturation, myelination, and brain activity patterns. *p*-Cresol, another tyrosine derivative and a metabolite, has also been directly associated with neurodevelopmental disorders.^239,240^ Further, certain bacteria-related metabolites, such as trimethylamine-*N*-oxide (TMAO), 5-aminovaleric acid (5-AVA), 5-AVA betaine (5-AVAB), imidazolepropionic acid, and hippuric acid have been reported to promote early-life axonogenesis both in vitro and in vivo.^241^ Moreover, the neurogenic properties of microbe metabolites may not be limited to early life given that indole, a tryptophan metabolite, has been reported to increase neurogenesis in the hippocampus of adult mice.^242^ Pilot studies using fecal microbiota transplantation in children enabled the assessment of whether early life interventions affecting the gut microbiota composition exerted long-term neurodevelopment effects.^243,244^ Thus, maternal fecal microbiota transplantation in cesarean-born infants was found to rapidly restore normal gut microbial development.^244^

Gut microbiota and its metabolites can affect the host metabolism of neuroactive compounds.^245^ The foremost examples of gut bacteria-derived neurotransmitters are aromatic amino acid derivatives dopamine and norepinephrine and glutamate derivative γ-aminobutyric acid.^246,247^ Microbiota have been found to extensively contribute to the levels of dopamine and norepinephrine via the activity of β-glucuronidase.^248^ Kynurenic acid, a tryptophan metabolite, functions as a glutamate modulator to reduce glutamate levels in the glutamatergic signaling in the hippocampus. Thus, enhanced cognitive abilities and memory in model animals have been achieved as a result of enhancing glutamate levels via limiting hippocampal kynurenic supply.^249,250^ Over 90% of serotonin in the body is known to be produced in the gut in a process in which gut microbes play an important regulatory role.^251,252^ Tyramine, deoxycholic acid, and 4-aminobenzoic acid have been reported to stimulate serotonin synthesis.^252^ Furthermore, microbiota-related metabolites, such as norepinephrine, indole, indole-3-aldehyde, isovaleric acid, butyric acid, and isobutyric acid stimulate serotonin release from enterochromaffin cells.^253,254^ Another gut microbiota metabolite with a likely connection to γ-aminobutyric acid (GABA) expression in brain is lactate.^255,256^ It has been shown to affect neural plasticity and has a beneficial effect on learning and memory in model animals.^257^ SCFAs, particularly butyric acid, may also have additional regulatory effects on the signal transduction to the brain via the vagal nerve and by inducing the biosynthesis of neurotransmitters in the CNS.^258^ Administration of SCFAs, such as butyric, acetic, and propionic acid, has been reported to improve stress response, anxiety, and depression.^259^ The presence of pipecolic acid in the CNS can be partially derived from the gut microbiota and has been also associated with GABA signaling and release.^245,260,261^

Gut microbiota has also proven vital for normal BBB function, especially during pre- and postnatal periods.^262^ In a mice model of traumatic brain injury typified by acute BBB disruption, sodium butyrate administration exhibited an alleviating effect on BBB integrity.^263^ Propionic acid, another SCFA gut microbiome metabolite, has also been shown to promote BBB integrity by mitigating oxidative and proinflammatory pathways.^264^ It has been suggested that the effects of SCFAs on BBB integrity may rather be brought about by peripheral signaling instead of direct uptake to the brain, as implied by animal models.^258^ Secondary bile acids, such as deoxycholic acid and ursodeoxycholic acid, may also modulate BBB integrity.^265,266^ Trimethylamine, a metabolite of dietary choline, betaine, and l-carnitine, has been reported to exert detrimental impact on the BBB integrity. It is noteworthy that physiologically appropriate doses of the oxidized form of trimethylamine, TMAO, improved the BBB integrity.^267^

A large portion of the brain’s energy production is consumed by the neurons, the major component of the nervous tissue, in order to maintain the excitability of the synapses.^268^ Lactate, a major gut microbiota metabolite, is known to augment neural activity as a primary energy source.^269^ Modulation of brain energy metabolism in the hippocampus along the GBA is suggested to be responsible for improvement in the cognitive function after intermittent fasting in model animals.^270^ It has been found that fasting considerably increased plasma levels of indolepropionic acid and tauroursodeoxycholic acid and fecal levels of SCFAs. Administrations of indolepropionic acid or tauroursodeoxycholic acid or a SCFAs mixture including acetic, propionic, and butyric acid have been able to reproduce the effects of fasting in cognition, hippocampal mitochondrial biogenesis, and energy metabolism-related gene expression. Findings connecting microbial metabolites to brain bioenergetics are preliminary but show that certain compounds are incorporated in the neuronal energy metabolism.

Gut microbiota metabolites reducing oxidative stress or neurotoxic proteins aggregation are functioning as neuroprotective agents. Metabolites that reduce inflammation or promote neurodevelopment or neurotransmission can also be considered as neuroprotectants. For example, ferulic acid is known to be metabolized by gut microbes.^271^ It exerts neuroprotective effects by reducing neuronal cell death and recovers memory deficits in a cerebral ischemia and reperfusion injury model.^272^ It has also ameliorated depressionlike behavior and oxidative stress.^273^ Dihydroferulic acid, a microbiota metabolite, has also been shown to exhibit neuroprotective antioxidative properties.^274^

**Figure 12:**
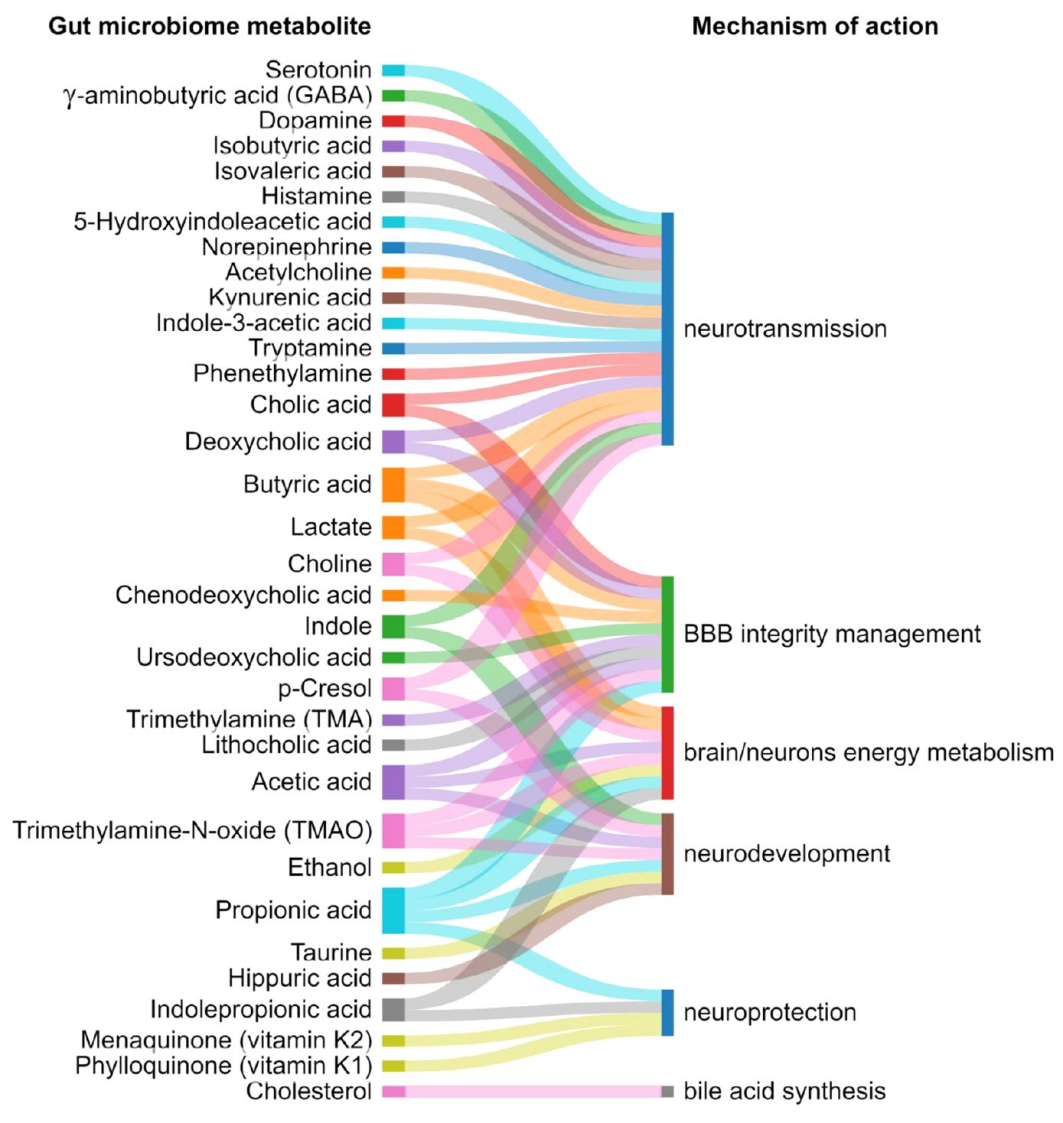
Exemplary gut microbiome metabolites and their mechanism of action in gut–brain communications.

The gut microbiome metabolites and their mechanism of action in mental health and brain development are depicted in Figure 12, and their function and associated diseases are summarized in Table 3.

**Table 3:** Gut Microbiota Metabolites, Their Function, and Associated Diseases

metabolite class/references	specific functions	associated diseases
short-chain fatty acids^258,285−290^	– gut microbiota composition regulation	– diabetes
	– gut barrier integrity support	– obesity
	– energy homeostasis support	– nonalcoholic fatty liver disease
	– gut hormone production	– ulcerative colitis
	– circadian rhythm regulation	– Crohn’s disease
	– proinflammatory cytokines inhibition	– colorectal cancer
	– immunomodulation	– autism spectrum disorder
	– water, sodium, calcium, magnesium absorption	– Parkinson’s disease
	– regulation of intestinal pH value	– diarrhea
		– IBS
		– constipation
		– functional dyspepsia (FD)
bile acids (BAs)^291−295^	– lipid and vitamin absorption regulation	– obesity
	– gut microbiota composition regulation	– nonalcoholic steatohepatitis
	– gut hormones production	– ulcerative colitis
	– intestinal immunity	– cancer
	– intestinal electrolyte and fluid balance	– multiple sclerosis
	– gut motility	– Alzheimer’s disease
	– gut barrier integrity	– Parkinson’s disease
	– lipid homeostasis	– traumatic brain injury
	– glucose homeostasis	– stroke
	– amino acid homeostasis	– amyotrophic lateral sclerosis
	– circadian rhythm	– IBS
	– neurotransmission	
tryptophan and indole derivatives^296−300^	– gut microbial spore formation	– ulcerative colitis
	– drug resistance	– Crohn’s disease
	– biofilm formation	– obesity
	– intestinal barrier function regulation	– stroke
	– gut hormone secretion	– mucosal candidiasis
	– gut motility	– autism spectrum disorder
	– immunomodulation	– Alzheimer’s disease
		– Parkinson’s disease
		– migraine
		– schizophrenia
		– IBS
choline metabolites^301−303^	– bile acid synthesis inhibition	– nonalcoholic fatty liver disease
	– inflammation promotion	– obesity
	– thrombosis	– diabetes
	– myocardial hypertrophy and fibrosis	– hypertension
	– mitochondrial dysfunction exacerbation	
vitamins^304−306^	– cellular metabolism regulation	– vitamin-associated diseases
	– immunomodulation	– schizophrenia
	– cell proliferation	– autism
	– vitamins supply	– dementia
		– IBS
		– IBD
neurotransmitters^307−309^	– gut motility regulation	– Parkinson’s disease
	– memory support	– autism spectrum disorder
	– stress response	– IBD
	– nervous system	– IBS
	– immune response	
lipids^184,310,311^	– systemic inflammation promotion	– diabetes
	– hyperinsulinemia regulation	– obesity
	– immunomodulation	– nonalcoholic fatty liver disease
	– bile acid synthesis	– hyperinsulinemia
		– hypercholesterolemia
		– chronic hepatitis C
gases^307,312−316^	– gut motility	– colitis
	– gut inflammation	– ulcer
	– epithelial secretion	– IBS
	– mucosal blood flow	

### Gut Microbiota Metabolites’ Role in Digestive System

**Figure 13:**
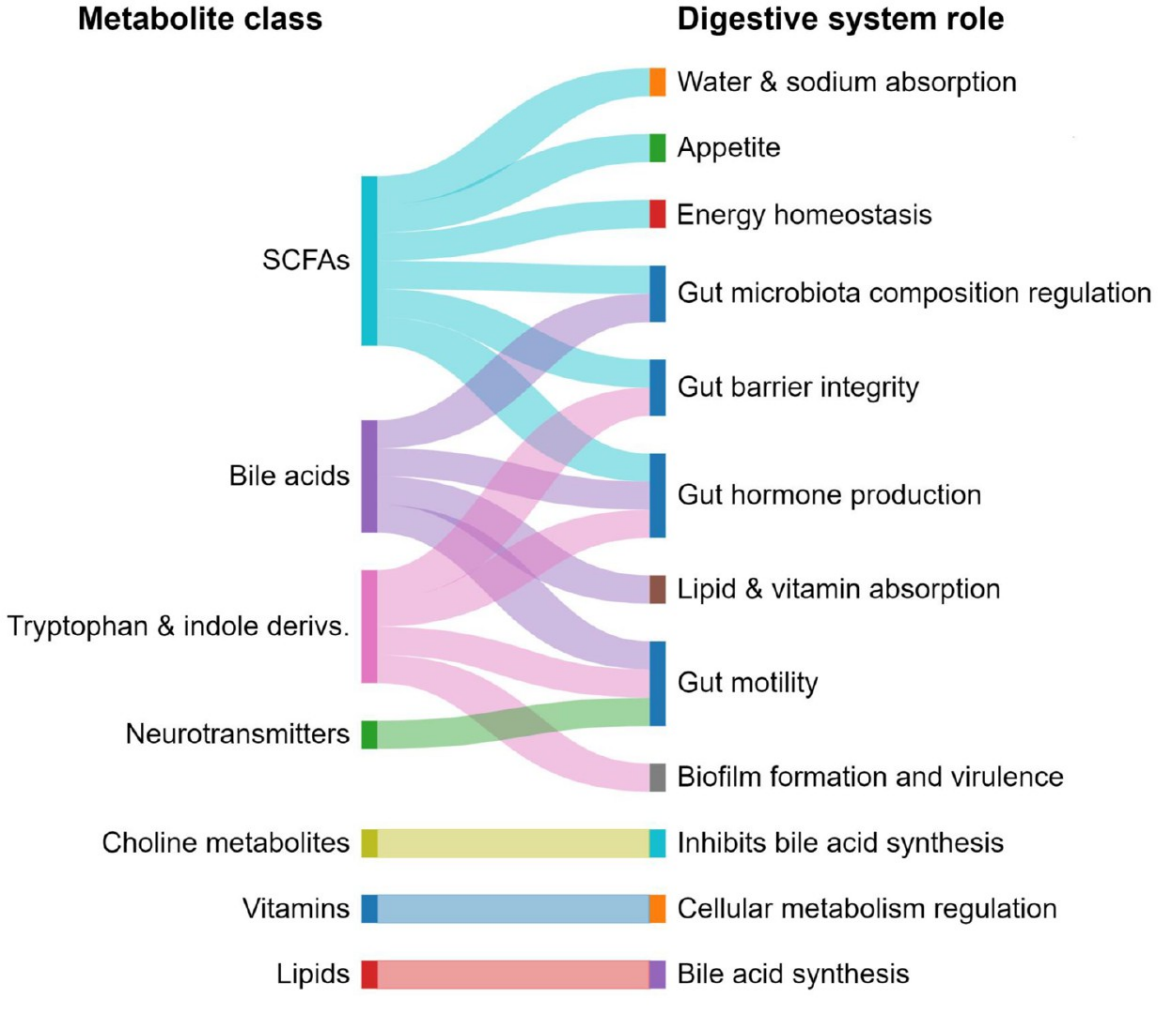
Gut microbiome metabolite classes and their roles in digestive system functions.

Gut microbiota imparts specific function in the host’s digestive system, in nutrient metabolism, xenobiotic and drug metabolism, in preservation of the integrity of the intestinal mucosal barrier, and in protection against pathogens (Figure 13).

Gut microbiota mostly get nutrients from dietary carbohydrates. Fermentation of the carbohydrates, including indigestible oligosaccharides by the microbes in the colon, such as *Bacteroides*, *Roseburia*, *Bifidobacterium*, *Faecalibacterium*, and *Enterobacter*, ends in the synthesis of SCFAs, such as butyric, propionic, and acetic acids, which are important sources of energy for the host.^275^ Gut microbiota have a positive role in lipid metabolism, as well, by controlling the lipoprotein lipase activity inhibition in adipocytes.^31^

Intestinal microbiota also exhibit a resourceful protein metabolizing machinery, which operates by means of the microbial proteinases and peptidases in conjunction with the human proteinases. Examples include the conversion of l-histidine into histamine by the bacterial enzyme histamine decarboxylase and glutamate to γ-amino butyric acid by glutamate decarboxylases.^276,277^

SCFAs can regulate the pH value in the intestine and regulate the absorption of water, sodium, calcium, and magnesium. Furthermore, SCFAs, especially butyrate, provide more than 70% of the energy for the intestinal epithelial cells on top of their abilities to inhibit the multiplication and growth of pathogenic bacteria and the activity of intestinal inflammatory mediators, thus playing an anti-inflammatory role in the intestinal tract.^278^

Lipid metabolites can affect intestinal permeability and intestinal immunity. The gut microbiota can produce lipopolysaccharides that could stimulate proinflammatory mediators, thereby disrupting the body’s immune system and inducing local and systemic inflammatory responses.^279^ Sphingolipids can be produced by the intestinal symbiotic bacteria Bacteroidetes and Prevotellaceae. It has been found in animal studies that sphingolipids can also aggravate intestinal inflammation.^280^

Indole-derived metabolites are produced by fermentation via *Clostridium sporogenes* and *Escherichia coli*. Such metabolites are able to participate in the regulation of gastrointestinal disorders by influencing the gut–brain axis and protecting against stress-induced damage in the gastrointestinal tract. Tryptophan is a key monoamine neurotransmitter involved in the regulation of central neurotransmission and intestinal physiological functions, and studies have shown that the gastrointestinal microbiome can regulate the gut–brain axis through tryptophan metabolism.^281,282^

Gases can be produced by gut microbiota as a result of the fermentation process. These gases include hydrogen (H_2_), methane (CH_4_), carbon dioxide (CO_2_), hydrogen sulfide (H_2_S), and nitric oxide (NO), which can modulate the gastrointestinal physiology of hosts.^236^

Xiao et al.^283^ in a recent review article highlighted the important microbial metabolites in the context of host physiology in patients with different IBS subtypes. The abundance of microorganisms and their corresponding metabolites in constipation-predominant IBS (IBS-C) and diarrhea-predominant IBS (IBS-D) differ, thereby providing a new avenue for the diagnosis and treatment of different IBS subtypes in the future. These microbiota-derived metabolites, such as bile acids (BAs), SCFAs, vitamins, amino acids, 5-HT, and hypoxanthine, can be produced directly by bacteria, or from dietary or relevant substrates. Fluctuations and alterations in the levels of metabolites produced by the host or microbiota provide insights into their interactions during IBS. Moreover, low levels of hypoxanthine may be associated with colonic epithelial energy and capacity for mucosal repair with hypoxia. Purine starvation has been identified as a potential novel mechanism underlying IBS with lower fecal hypoxanthine abundance in IBS-C and IBS-D. Additionally, mucosal biofilms are an endoscopic feature of IBS and are associated with bacterial and BA metabolites dysbiosis. Additionally, deficiency in levels of both vitamins D and B6 have emerged as causative factors in IBS symptoms pathogenesis.^283^

Microbial dysbiosis and metabolites derived from interaction of the host and gut microbiota have been reported as an intermediate link contributing to the development of functional constipation via various signal pathways, including but not limited to SCFAs, BAs, and methane that occupied a more important position.^153^ 5-HT is also involved in the modulation of gut motility and secretory, as well as sensory, transmission in patients with constipation.^284^ Altogether, current studies have provided us with a new conception on the microbial mechanisms and therapeutic targets of constipation.

The gut microbiome metabolites and their function and associated digestive diseases/disorders are summarized in Table 3.

## Gut Microbiome–Brain Axis

CNS and the human GI tract communicate through the gut–brain axis (GBA). This bidirectional connection involves neuronal, endocrine, and immunological mechanisms. The gut is considered as our “second brain,” because of its hosting the enteric nervous system (ENS), a neural network that allows the gut to work without instructions from the brain. The ENS maintains control of our digestive system; it plays an important role in peristalsis, secretion, and pain perception. There is mounting data that gut microbiota are the source of a number of neuroactive and immunocompetent substances, as shown above, that help to shape the structure and function of brain regions involved in the control of emotions, cognition, and physical activity and contribute to the proper maintenance of gastrointestinal homeostasis. Most GI diseases are associated with altered transmission within the GBA that are influenced by both genetic and environmental factors.^317^

Functional gastrointestinal disorders (FGIDs) were previously considered as purely functional disorders with no scientific confirmation of a clear pathogenetic mechanism. According to Rome IV, the phenotype of FGIDs results from an altered transmission of nerve and biochemical signals within the gut microbiome–brain axis with mechanisms controlled by both genetic and environmental factors. Consequently, FGIDs were recently renamed into disorders of gut–brain interactions.^318^ The overlap of DGBI and CNS disorders has been documented, and it has been demonstrated that approximately one-third of IBS patients suffer from depression. It is estimated that psychiatric symptoms occur in at least 36.5% of FGIDs patients. Stasi et al. found that the highest prevalence of mental or spectrum disorders is in patients with functional constipation (60%) compared with patients diagnosed with FD (52.4%), IBS (36.5), and/or functional bloating (47.6%). The most prevalent psychiatric disorders observed in FGIDs were general anxiety disorder and panic.^319^

Recent advances in this field have enabled us to better understand some of the pathophysiological consequences of an aberrant reciprocal gut–brain network, including exacerbated gut inflammation disorders, altered responses to stress, as well as altered behavioral states. Therefore, the GBA presents an attractive target for the development of novel therapeutics for an ever-growing list of disorders related to mental and digestive health. Improved targeting of the gut microbiome–brain axis, for example through application of biotics, is expected to pave the way for the development of novel disease therapies and self-care products to promote and maintain heathy status.^320^

### Irritable Bowel Syndrome

IBS is one of the most common DGBI worldwide and typically presents in early adulthood with symptoms including abdominal pain, bloating, and altered bowel habits. On the basis of the bowel habits, IBS can be classified into four subtypes: constipation-predominant, diarrhea-predominant, mixed-type (IBS-M), and undefined IBS (IBS-U). Symptom intensity varies over time and between individuals, but IBS has been reported, in severe cases to affect quality of life as much as renal impairment or diabetes.^321^ IBS represents up to 50% of all referrals to gastroenterologists with a prevalence rate of up to 11% globally.^322^ The recent consensus view is that IBS results from abnormal gut–brain interactions. Recent epidemiological data has suggested that in individuals developing both IBS and psychological features, the former preceded the latter in two-thirds of cases, and the latter preceded the former in one-third.^323^ IBS is associated with abnormalities of central pain processing but also increased gut permeability, mast cell activation, disordered motility, and dysbiosis.

A recent genome-wide analysis study for 53 400 IBS patients and 433 201 controls highlights shared genetic pathways between IBS and mood and anxiety disorders. The study identified and confirmed six genetic susceptibility loci for IBS, and four of them are associated with mood and anxiety disorders, thereby suggesting, for example, that shared pathogenic pathways rather than anxiety cause abdominal symptoms.^324^

5-HT signaling is one particular pathway of importance in IBS pathogenesis. It has been demonstrated that a functional GI tract involves 5-HT signaling between enterochromaffin cells acting as sensory transducers, and the majority of 5-HT is synthesized, stored, and released by these cells, which interact with intrinsic and extrinsic sensory nerve afferents in the mucosal layer of the gut.^320^ 5-HT signaling controls many GI functions, including secretion; vasodilation; peristalsis; and sensory perception, such as pain and nausea.^325−327^ Moreover, serotonergic function and tryptophan metabolism are known to be altered in IBS patients.^328−331^

IBS pathophysiology implicates altered gut microbiota composition, impaired intestinal mucosal integrity, and low-grade inflammation. In addition to pathways through the circulatory system, several of these factors may also trigger fluctuations in the activity of the ENS with subsequent effect on the brain.^332^ Furthermore, the vagus nerve can be modulated by diet-responsive gut microbes and metabolites, such as short-chain fatty acids, or endocrine factors, enzymes, and neurotransmitters, such as serotonin, dopamine, acetylcholine, glutamate, γ-aminobutyric acid, and noradrenaline.^333−336^ Each of these factors are potentially affected by alterations in microbiota composition and are involved in IBS pathology.^337^

Identifying a clear IBS microbial signature is not an easy task because of the heterogeneity of the healthy gut microbiota. However, multiple studies have shown differences in the gut microbiota between IBS and healthy controls. A recent systematic review showed that IBS patients have increased levels of the bacterial families Enterobacteriaceae, Lactobacillaceae, and Bacteroidales, whereas *Bifidobacterium*, *Faecalibacterium*, and *Clostridiales* were decreased compared with healthy controls.^122^ On the contrary, Hugerth et al. recently reported no distinct microbiota signature of IBS in a random Swedish population of 3556 participants.^338^ Interestingly, intestinal bacterial composition has been reported to be highly dependent on sample type and regional localization. Also, mucosa-associated bacterial composition of the sigmoid colon differs between patients with IBS and healthy controls.^339^

One of the most consistent findings in brain neuroimaging of IBS patients has been alterations in the structure and function of key regions of the somatosensory network, including the globus pallidus, putamen, and caudate, which composes the basal ganglia.^340^ Increased gray matter density in the hypothalamus and decreased gray matter density in the prefrontal cortex have been reported in the IBS brain.^341^ In rectal distention experiments, patients with IBS had a differential brain response in the pain matrix and default mode network.^342^ IBS patients showed increased engagement of endogenous pain faciliatory pathways and decreased levels of the endogenous pain inhibitory mechanism in the brain regions associated with visceral afferent processing and emotional arousal, including the left dorsal anterior cingulate gyrus and the bilateral anterior insulae.^343^ A meta-analysis of adult studies that evaluated brain response to rectal balloon distension by functional MRI (fMRI) reported differences between healthy control subjects and patients with IBS in these brain regions.^344^ IBS patients with a history of abuse reported increased pain and anxiety with rectal distension accompanied by similar fMRI changes.^345^ The stress and arousal circuit demonstrated in human subjects by fMRI shares significant homology with the stress circuit related to CRF–CRF1 receptor signaling in rodents, thereby potentially implicating the HPA axis as a facilitator of gut–brain axis communication.^346^

Recently, evidence for disrupted subcortical and cortical regions mediated by gut microbial modulation has been emerging in IBS. An association between brain region-to-region functional connectivity and microbiota has been reported. Labus et al. found a correlation between Clostridia and Bacteroidia with connectivity of the thalamus, the basal ganglia (caudate nucleus, putamen, pallidum, nucleus accumbens), the superior part of the precentral gyrus, the anterior insula, and the ventral prefrontal regions in IBS patients.^347^ Recently, the same group also reported on fecal metabolites and resting state fMRI where the differences in histidine, cysteine, glycine, glutamate, spermidine, and anserine were significantly associated with the alteration in the left dorsal part of the posterior cingulate gyrus to the left putamen. Also, the changes in histidine, tryptophan, uracil, 2-deoxyuridine, thymidine, and succinate were differentially associated with the alteration in the right superior frontal gyrus to the right putamen. Interestingly, this interaction may be mediated by aberrant tryptophan signaling in IBS, which is important because it is a substrate for serotonin synthesis.^348^

Previous studies have compared brain differences between IBS-C and IBS-D. These studies have examined task states and have shown group-related differences in brain networks involved in integrating emotions [emotional arousal network, (EAN)], perception [sensorimotor network, (SMN); salience network, (SAL)], visceral functions [central autonomic network, (CAN)], and pain processing [default mode network, (DMN); central executive network, (CEN); SMN; SAL; EAN; and others).^349^ Prior studies comparing IBS-C, IBS-D, and healthy controls (HCs) undergoing aversive rectal stimuli have identified abnormal connectivity in the SAL^350,351^ and EAN^343,350^ in IBS-C and in the occipital network (OCC)^351^ in IBS-D. This may indicate a greater importance of alterations in sensory, emotional, and autonomic responses associated with the perception of visceral pain and discomfort in IBS-C. A recent study has tested the hypothesis that IBS-C exhibits bowel-habit-specific changes in the brain that reflect altered sensory and emotional regulation processing of visceral inputs from the “top-down,” while IBS-D would have widespread gut microbiome and metabolome changes (e.g., tryptophan, SCFAs), which may translate to “bottom-up” brain changes (e.g., SMN, DMN).^349^ Indeed, in IBS-D, the study’s findings showed a correlation between high levels of gut metabolites tryptophan and phenylalanine and aberrant connectivity in brain regions involved in processing unpleasant visceral stimuli (SMN) and self-related thoughts (DMN).^349^ These results suggest that increased tryptophan in the gut may lead to loosened stool, and tryptophan-related signaling may travel to the posterior insula and increase pain perception and emotional salience in IBS-D, thereby suggesting a “bottom-up” signaling direction. However, IBS-C’s microbiome and metabolome resembled HC, and the increased connectivity in the default mode (DMN) and salience (SAL) networks compared with IBS-D may indicate abnormalities in the emotional physiological processing of visceral signals.^349^ IBS-C’s relatively isolated brain changes may indicate a more “top-down” mechanism to produce the constipation-predominant phenome. That study by Sarnoff et al. has shown a link between the chronicity of IBS symptoms, *B. stercoris* and *F. prausnitzii*, and brain connectivity in the caudate nuclei.

Thus, we might be in the mere beginning of understanding how alterations in gut microbiota may lead to the disruption of the intricate host–gut–microbiota interaction: is it a cause or a result of IBS pathology? In the past decade, much knowledge has been gained from clinical microbiota-altering interventions, such as the low-FODMAP diet and fecal microbiota transplantation (FMT), which have emerged as debatably successful treatment strategies. However, their effects on the gut microbiome–brain axis are still far from understood. It has been proposed that probiotic amelioration of IBS symptoms may be acting indirectly through an anti-inflammatory mechanism.^352^ Such anti-inflammatory mechanisms may also be partially responsible for the positive effects of dietary restrictions, such as those seen in the low-FODMAP (fermentable oligosaccharides, disaccharides, monosaccharides, and polyols) diet in IBS.^353−355^ An alternate method for IBS treatment involves FMT from non-IBS individuals to patients with IBS.^356−359^

### Functional Dyspepsia

Functional dyspepsia (FD) is a worldwide prevalent DGBI affecting 10%–30% of adults and 3.5%–27% of children worldwide.^360^ The main clinical symptoms of patients are early satiety, postprandial discomfort, epigastric pain, epigastric distension, epigastric burning, loss of appetite, belching, nausea, and vomiting, which are often accompanied by anxiety and depression.^361^ FD pathogenesis is linked to GI dysmotility, visceral hypersensitivity, impaired gastric tolerance, disrupted gastrointestinal mucosal integrity, abnormal function of the gut microbiome–brain axis, increased eosinophils in duodenum, dysbiosis, *Helicobacter*-pylori infection, postgastrointestinal infection, diet, genetics, and mental and psychological factors.^278^

An increasing number of studies have confirmed the close association between disturbance in the relative abundance and composition of the gastrointestinal microbiota and the occurrence and progression of FD. *Actinomyces*, *Atopobium*, *Leptotrichia*, *Prevotella*, and *Veilonella* counts differ between FD and control patients.^362^ The finding was preceded by an observation that, in FD patients, gut barrier integrity is impaired and expressed as lowered transepithelial resistance; diminished expression of proteins of tight junctions; and lastly, elevated levels of mast cells, eosinophils, and interstitial lymphocytes.^363^

Surprisingly, FD patients not only had different gastrointestinal microbiota compared with non-FD, but also had different oral microbiota abundance and composition. Proteobacteria were the dominant bacteria in FD patients’ saliva, while Bacteroidetes were the dominant bacteria in healthy controls. The abundance of Spirochaetes in FD patients was higher than that in healthy controls, while the abundance of Fusobacteria, TM7, and Proteobacteria was lower than in healthy controls, and the levels of *Kingella* and *Abiotrophia* genus levels were also significantly different.^364^

Mental illness plays a significant role in the pathogenesis of FD. Anxiety at baseline has been shown to increase the risk of developing FD by almost 8 times after 10 years follow-up.^365^ Interestingly, multiple studies highlighted that the prevalence of anxiety and depression is significantly increased in patients with FD compared with healthy people. Furthermore, pathophysiological research indicates that psychosocial factors and mental disorders may play a role in FD by modulating both visceral signal processing in the brain^366^ and the effects of stress hormones on pain perception.^367^ Furthermore, it is known that psychosocial factors and stress hormones also affect other aspects of the GI tract, such as motility, immune system activation, permeability, and microbiota.

However, FD symptoms are thought to induce anxiety or depression because of a cytokine response in low-grade intestinal inflammation, which plays an important role in the development of psychological distress in patients with FD.^368^ A growing body of evidence suggests that the gut microbiota communicates with the central nervous system, possibly neuro-immuno-humoral pathways, thereby influencing brain function.^369^ Furthermore, microbiota release neuroactive compounds, such as GABA, serotonin, dopamine, and acetylcholine, thereby acting locally on the enteric nervous system. Some of these neuroactive substances access the brain through the blood and the circumventricular organs or via the vagus nerve. Therefore, it could be hypothesized that a disturbed microbiome might affect mental health, followed by anxiety and depression. Thus, the mental disorders might be a consequence of the dysbiosis and, therefore, promote the development of FD, which may explain the findings that anxiety increases the risk of FD^365^ and observations indicating that psychosocial factors and mental disorders may play a role in FD by modulating visceral signal processing in the brain.^366,370^

### Functional Constipation

Functional constipation (FC) is a common DGBI with a global prevalence ranging approximately from 10.1 to 15.3%^371^ and is characterized by difficult bowel movements and/or a sense of incomplete evacuation, thus influencing quality of life.^372^ Previous studies showed that the gastrointestinal microbiota composition of constipation is clearly distinct from that of normal individuals. The species diversity of microbiota in the patient samples was lower than that in healthy subjects; it was also accompanied by significantly reduced levels of *Bifidobacterium* and *Lactobacillus* and an increased abundance of Desulfovibrionaceae.^373^ Levels of butyrate-producing bacteria, such as *Faecalibacterium* and *Roseburia*, were significantly reduced in patients with FC.^373^ It has also been confirmed that the relative abundance of methanogenic bacteria is increased in patients with slow transit constipation relative to healthy subjects.^373,374^ In another study, Chen et al. collected 3056 fecal amplicon sequence data from five research cohorts and used machine-learning methods to construct the constipation discriminant model. The model identified 15 top-ranking biomarkers, particularly inflammation-related pathogenic bacterial genera *Serratia*, *Dorea*, and *Aeromonas*.^375^ A recent shotgun metagenomics study confirmed the results of previous studies by showing that the relative abundance of *Roseburia intestinalis*, a prominent butyrate-producing bacterium, was reduced in patients with constipation in comparison with healthy controls, and the microbiome corresponding to constipation was enriched for pathways implicated in methanogenesis.^376^ In contrast, the microbiome of healthy individuals was characterized by high levels of genes associated with carbohydrate, fatty acid, and lipid metabolism.^150^

Notably, different intestinal sites harbor certain gut microbiomes, yet the majority of recent research has focused on the analysis of fecal-derived microbiota, which are accessible via noninvasive sampling methods. However, luminal microbiota is generally considered to be representative of the distal large intestinal content. The mucosa-associated microbiota, which live in more intimate contact with the host, cannot be fully replicated by fecal microbiota.^377^ In patients with constipation, there is even less similarity between fecal- and mucosa-associated microbiota compared with healthy controls and patients with diarrhea.^116^ These differences may be due to drier stool allowing fewer signaling molecules to enter the mucosa^378^ or the longer transit time providing more opportunities for the communities to diverge.^116^ Hence, mucosa-associated microbiota are more likely to affect the host’s epithelial and mucosal function than luminal microbiota.^379^ Comparative analyses between fecal and mucosal microbiota showed that the colonic mucosal microbiota composition was correlated with constipation (and was accompanied by a significant increase in Bacteroidetes), while the fecal microbial communities were correlated with colonic transit and methane production rather than constipation.^380^ However, more evidence is needed to prove the relationship between the mucosal profile and constipation.

Slow gut transit has been associated with reduced fecal water content, higher fecal pH, higher microbial cell density and diversity, and a shift in microbial metabolism from saccharolysis toward proteolysis, as reflected by reduced levels of short-chain fatty acids and increased levels of branched-chain fatty acids (BCFA).^381^ It is likely that once easily accessible carbohydrate sources become scarce in the colon, the gut microbes switch to ferment dietary and mucin-derived proteins. While saccharolysis by the gut microbiota gives rise to SCFA that are beneficial for the host and a source of energy for the colonocytes, proteolysis can lead to the accumulation of compounds such as BCFA, phenols, indoles, ammonium (NH_3_) and hydrogen sulfide (H_2_S) that are generally considered detrimental for health. Moreover, hydrogen (H_2_) with carbon dioxide (CO_2_) or formate can be converted into methane (CH_4_) by methanogenic archaea, which are also linked to slower transit time. In addition, the production and circulation of secondary bile acids and hydrolysis of host-derived glucuronides excreted via bile can also be affected by alterations in gut transit time.^381^

FC in patients would accompany mental disorders like anxiety and varying severity of depression.^382^ Evidence from recent neuroimaging studies illustrated that FC patients had significant structural and functional alterations in brain regions that are involved in visceral sensorimotor, cognitive control, and emotional regulation,^383−389^ thereby confirming its reclassification as DGBI. Among these altered brain regions in FC patients is the anterior insula, which is generally regarded as a critical node for its essential role in processing interoceptive signals, modulating visceral activities, and regulating emotions and cognitions.^383,385,389−391^

### Stress and Stress Resilience

Chronic stress is rapidly becoming a global societal challenge. Stress constitutes a state of threatened homeostasis triggered by intrinsic or extrinsic adverse forces (stressors) and is counteracted by an intricate repertoire of physiological and behavioral responses aiming to maintain/reestablish the optimal body equilibrium (eustasis). Stress is a nonspecific response of the body to any demand imposed upon it that disrupts the body homeostasis and manifests with symptoms such as anxiety, depression, or even headache.^392^ Stress can be hardly avoided in the present-day, modern, competitive life. Although eustress is important for people’s rapid reaction to threats, chronic stress is associated with detrimental effects on physical health and adverse implications on the immune, neuroendocrine, and central nervous systems.^392^

Acute stress activates the HPA axis, thereby resulting in an immediate release of cortisol. This response prepares the individual to defend against or escape from a threat. After the threat subsides, normal homeostasis should return. However, when that fails to occur, chronic activation of the stress response results in dysregulation of the HPA axis and an increased risk of subsequent diseases/disorders.^320^ It also acts on the gut to increase the release of proinflammatory mediators, which leads to increased gut permeability.^393^ Repeated exposure to stress can initiate a vicious cycle of low-grade inflammation and negatively impacts the intestinal barrier and immune signaling within the gut.^394,395^

A link between stress and the abundance of lactobacilli in mice was discovered for the first time more than 40 years ago.^396^ Several preclinical studies have documented that stress impacts gut microbial composition in a number of different hosts using different stress models ranging from water avoidance to maternal separation, heat, and acoustic stress and overcrowding.^320^ These results have shown clinical relevance and translated into human studies, thereby showing the influence of stress on gut microbiota and gut microbiota on stress modulation through different stressors, such as surgical intervention, academic examination, or military training, among others.^397−400^

Maternal stress during pregnancy displays a distinct fecal microbiota profile, which has generational consequences. The maternal microbiota influences offspring microbiota and correlates with hyper-reactivity of the HPA axis, together with other perinatal factors, as a key determinant of offspring outcomes. These findings have been confirmed in humans in a population-based study whereby infants born to mothers with high cumulative stress during pregnancy exhibited an aberrant microbial composition.^401^

The effects of early life stress on the microbiota may extend to adulthood.^49^ It is, therefore, plausible that changes in the gut microbiota due to stress at least partially mediate the onset of stress-related depressive or anxious episodes. Correlational studies have shown that fecal microbiota composition in individuals with anxiety or depression differs from that in healthy controls.^202,402,403^ Women with a higher fecal *Prevotella* abundance experienced increased negative emotional response to viewing negative images and lower brain activity in the hippocampus than those with a higher *Bacteroides* abundance.^404^

Stress resilience is the ability to experience stressful events without the development of chronic elevated stress (psychological and/or biological) and associated changes in emotional behavior.^405,406^ Stress susceptibility is related to psychological factors, such as passive coping skills and high emotional reactivity, but is also associated with biological factors such as hypo- or hyper-responsiveness of the stress response system, sex hormones, central and peripheral immune activation, and glucocorticoid resistance.^407^ The gut microbiome is a biological factor that is emerging as a possible influencer of stress resilience. The broad influence of the gut microbiota on human health, including psychiatric health, has begun to be realized and understood over the past decade. Preclinical studies have reinforced this principle showing a connection between the gut microbiome–brain axis and stress resilience. Li et al. recently reported that certain mice exposed to chronic stress were found to be resilient to stress-induced corticosterone and anxiety-like behavior. These mice contained a relative abundance of *Lactobacillus* species within their gut microbiome. Subsequent stress-susceptible mice saw decreased anxiety-like behavior and corticosterone levels with *Lactobacillus murinus* supplementation.^408^

Modulation of the gut microbiome has emerged as a possible way to improve stress resilience and mental health. In 2013, Dinan et al. coined the term psychobiotics, which refers to live microorganisms when ingested in adequate amounts that produce a health benefit in patients suffering from psychiatric illness; the definition has been expanded to include other interventions that modulate the gut microbiome, such as prebiotic.^409^ The term psychobiotics has since been widely adopted by neuroscientists conducting research on neurodegenerative diseases and depressive disorders in order to describe the use of different biotics to tackle depression, stress, anxiety, and other mental health complaints through the GBA.

### Sleep

Adequate sleep quality and sufficient duration are necessary to support both mental and physical health and overall quality of life.^410^ Inadequate sleep in either duration and/or quality has been increasingly recognized as a global public health issue. Sleep disturbances are typically characterized by a decrease in one’s ability to initiate and maintain sleep and by a reduced proportion of the deeper, more restorative sleep.^411^ Increased risk of developing chronic diseases, such as obesity, type 2 diabetes, heart disease, some types of cancer and mental illness, has been associated with inadequate sleep.^411,412^

Evolving evidence has shown the impact of gut microbiota on sleep. In humans, previous research has shown that partial sleep deprivation can alter the gut microbiome composition in as little as 48 h;^413^ however, longer periods of sleep deprivation apparently do not have this effect.^414^ A more recent study showed that high sleep quality was associated with a gut microbiome containing a high proportion of bacteria from the Verrucomicrobia and Lentisphaerae phyla and that this was associated with improved performance on cognitive tasks.^415^

Microbiome diversity was positively correlated with sleep efficiency and total sleep time and was negatively correlated with the sleep fragmentation, thereby indicating that diversity of the gut microbiome promotes healthier sleep. However, two previous studies in humans suggested that microbiome diversity is insignificantly affected following a period of sleep restriction.^413,414^ A critical difference between these studies is that the former study measured sleep over an extended period of time (one month), while the latter two studies manipulated sleep by experimentally restricting sleep. Accordingly, it is possible that short-term manipulations to sleep do not influence the gut microbiome diversity, but rather that microbiome diversity can influence sleep in the long term.

Gut microbiota may affect sleep status via degradation products, such as muramyl peptides (MPs), lipopolysaccharide, and melatonin.^416^ These degradation products could activate immune cells that lead to the release of cytokines, which could affect sleep. Cytokines represent a potential critical interface between sleep physiology and gut microbiome composition. The acute phase pathway, cytokines IL-1β and IL-6, in particular, are strongly associated with sleep physiology. IL-1β is a major somnogenic factor.^416^ IL-1β administration in human and nonhuman animals increases spontaneous sleep and fatigue, and IL-1β increases with ongoing sleep loss.^416^ Unlike IL-1β, IL-6 is not a direct somnogenic factor, but sleep loss results in increased IL-6 levels.^417^ In the gut, IL-6 and IL-1β-mediated inflammation fluctuates in response to stress and disease.^418^ For example, intestinal mucositis results in an increased expression of IL-6 and-IL-1β in the small intestine^419^ and in serum and colon tissue^420^ in mice. In humans, chronic stress, alone, increases IL-6 and-IL-1β.

Alterations in the microbiome have been shown to influence neurotransmission of serotonin in both the peripheral and central nervous system.^411^ While this may convey a positive impact on mood and psychological well-being,^421^ it also has the potential to influence sleep^422^ as serotonin is acetylated and, then, methylated to yield melatonin—the hormone important in helping regulate sleep/wake cycles.^423^

A recent meta-analysis involving 36 studies showed that sleep disorders were common in IBS, and the prevalence rate was 37.6% (95% confidence interval: 31.4% to 44.3%).^424^ The pooled odd ratio revealed that sleep disorders were significantly associated with IBS. The reason why sleep disorders are associated with IBS remains unclear; however, the gut microbiome–brain axis could play an important role in the pathogenesis of both. Modification of the autonomic nervous system activity has been observed in cases of sleep deprivation, which indicates that sleep disorder might be associated with autonomic dysregulation.^425,426^ It has been postulated that sleep inhibits the HPA axis, and sleep disorder may result in a 24 h increased secretion of cortisol.^427^ Moreover, IBS symptoms, such as abdominal pain, may activate the sympathetic nervous system and, hence, reduce sleep efficiency.^428^ Microbiome modulation has been shown to influence melatonin production and modulate IBS symptoms in individuals with a normal circadian rhythm.^429^ Overall, although the reason for sleep disorders seen commonly among IBS patients is obscure, a gut microbiome–brain axis disorder may underlie this association.^427^

An additional layer of evidence has been shown by the intertwined interactions between the gut microbiome and the central and peripheral circadian rhythms.^430^ The disruption of the host circadian rhythm alters the gut microbiome equilibrium. In addition, the microbiome is able to mediate host clock gene expression in peripheral organs and the suprachiasmatic nucleus.

Intestinal bacteria have shown inherent circadian rhythms, as shown in previous metagenomic studies.^430^ Diurnal fluctuations in abundance and activity have been observed in Clostridiales, Lactobacillales, and Bacteroidales, which account for ∼60% of the microbiota.^431^ Studies on human stool samples of *Enterobacter aerogenes* have demonstrated responsivity to the circadian hormone melatonin, as well as a daily rhythm.^432^ The gut epithelium experiences differential bacterial species and metabolites depending on the time of day and expresses toll-like receptors that sense microbiotal metabolites in a rhythmic pattern.^433^ It has been proposed that the gut microbiome influences the rhythmic expression of the host’s internal clock by signaling molecules, such as butyrate, and by oscillations in microbiotal bacterial content in response to feeding patterns.^434^

### Cognitive Function

Abundant evidence supporting the role of the gut microbiota in modulating cognitive function is mostly based on animal research. However, few studies have examined the influence of gut microbes on human cognition and supported the clinical relevance. One of these studies showed that the gut microbiota composition of obese and nonobese subjects was linked with scores in speed, attention, and cognitive flexibility coupled with alterations in neural activity in the thalamus, hypothalamus, and amygdala, thereby suggesting that obesity affects the microbiota composition and subsequent cognitive performance.^435^ Additionally, the microbiota composition in 1-year-old babies was associated with cognitive development. Three groups of microbial composition have been identified where better performance was seen in the group with higher levels of *Bacteroides*.^436^ This group was also less likely to be born via C-section, which supports the previous observation linking delivery mode with child cognitive development,^437^ thereby highlighting the importance of gut microbiota colonization in cognitive development and function.^436^

Microbiome modulation has demonstrated beneficial effects on cognitive performance. Lactobacillus strains have improved cognitive performance in healthy elderly subjects.^438^ Fermented milk product supplemented with a probiotic has been shown to modulate the activity of brain regions involved in cognitive performance during an emotional attention test in healthy women.^439^ Also, the modulation of the microbiome via inulin prebiotic has been shown to improve memory and mood in healthy individuals.^440^

These results taken together indicate the potential role of the gut microbiome–brain axis in regulating cognitive performance and that microbiome modulation could be a promising approach for improving cognitive function in both healthy and vulnerable individuals. However, much more work is needed to understand why specific microbiome-related interventions have the potential to modulate cognition.

### Emotional Well-being

Gut microbiota is emerging as a key mechanism for modulating emotional well-being.^441^ In fact, emotional disorders, such as depression and anxiety, are frequently accompanied by functional gastrointestinal disorders, which suggests an association between gut function and psychiatric diseases.^442,443^ Recent research reveals the gut–brain axis association with the vagus nerve plays an important role in emotional well-being. The subdiaphragmatic vagus nerve is a major modulatory pathway between the brain and gut microbiota.^444^ Data suggest that fecal microbiota from depressed mice produce depression-like phenotypes and abnormal gut microbiome composition when transplanted to nondepressed mice via the subdiaphragmatic vagus nerve.^445^

Gut microbiota is emerging as a key mechanism for the modulation of emotional well-being.^441^ In fact, emotional disorders, such as depression and anxiety, are frequently accompanied by functional gastrointestinal disorders, which suggests an association between gut function and psychiatric diseases.^442,443^ Findings from a longitudinal study further suggest that intestinal infections significantly predict the future onset of anxiety disorder.^446^

Evolving clinical evidence indicates the significant links between the gut and emotion; for example, altered gut microbiota composition^447^ was reported in patients with depressive disorder in terms of fecal microbial diversity, as well as the level of the genus *Faecalibacterium*.^403^ In healthy adults, self-rated higher quality of life and favorable personality types (high in openness and conscientiousness) were associated with the composition of certain gut microbiota (i.e., *Faecalibacterium*, *Coprococcus*, and Lachnospiraceae), as well as an enriched diversity of the gut microbiota community.^448,449^

Recent studies suggest that enterotypes play a role in regulating the association between the gut microbiome and mental health.^404,449^ Enterotypes refer to robust stratified clusters on the basis of the variation found in the levels of one of three genera in the gut: *Bacteroides* (enterotype 1), *Prevotella* (enterotype 2), and *Ruminococcus* (enterotype 3).^450^ Individuals’ enterotype cluster depends on, in part, long-term diet—i.e., the amount of ingested animal protein/saturated fats (*Bacteroides*-type) versus carbohydrates/simple sugars (*Prevotella*-type)—whereas they are less influenced by the hosts’ body mass, age, and sex.^450−452^

A recent brain imaging study found distinct patterns of the emotional process and brain connectivity between stratified enterotypes; clusters with a greater abundance of *Prevotella* show higher levels of emotional response, along with prominence in the connectivity of emotional, attentional, and sensory-processing brain regions when compared with *Bacteroides*-dominant clusters.^404^ A large-scale microbiome study also revealed that the *Bacteroides*-enriched enterotype is significantly associated with a lower score on the subjective feeling of quality of life, as well as a higher score regarding depressive symptoms.

A recent exploratory study revealed that gut microbiome diversity is related to emotional well-being and that enterotypes significantly moderate the links between emotional well-being and gut microbiome diversity.^441^ The enterotypes did not alter mood status, itself, but moderated the strength of the association between one’s mood and gut microbiome diversity. In the *Prevotella*-dominant group, emotional status was more closely related to gut microbiota diversity, such that a positive effect was associated with increased gut microbiota diversity. However, in the *Bacteroides*-dominant group, one’s mood status was not significantly associated with gut microbiome diversity. This finding is in line with the results of Tillisch et al.^404^ in that only the high-*Prevotella* group displayed an increased response to affective images in the limbic system. Such findings suggest a significantly tighter connection between emotional well-being and the well-being of the gut microbiota community, particularly in the *Prevotella*-dominant condition.

There is an evolving body of evidence suggesting that the modulation of the gut microbiome by biotics administration or via food supplements (i.e., psychobiotics) may closely affect one’s mood. Benton et al.^421^ showed that the consumption of probiotic-containing yogurt improved the self-reported mood of those whose mood was initially poor. Messaoudi et al.^176^ showed that consumption of the probiotics reduced anxiety and depression scores in subjects with reduced urinary free cortisol. Also, consumption of *Lactobacillus helveticas* and *Bifidobacterium longum* reduced somatization, depression, anger and hostility, hospital anxiety, depression-scale global scores, and self-blame scores on coping checklists and increased focus on problem solving, but there was no effect on perceived stress.^176^ Moreover, the administration of a combination of *Lactobacillus acidophilus*, *Lactobacillus casei*, and *Bifidobacterium bifidum* for 8 weeks improved the depression score.^453^ In a 2017 systematic review by Wallace and Milev of 10 clinical trials, most of the studies found positive results on measures of depressive symptoms.^454^ A recent meta-analysis^455^ included 30 randomized placebo-controlled studies and revealed that most probiotics did not affect mood, anxiety, depression, and psychiatric distress when compared with placebo at the qualitative questionnaire level; however, on the quantitative meta-analysis level, probiotics intervention showed slightly significant effect compared with placebo. In addition, EEG and imaging studies summarized in a recent review proved that probiotics are able to exert effects on CNS function in humans; although, the number of studies is still low.^455^

Overall, there is emerging evidence on the link between one’s emotional status and gut microbiome diversity and composition. The current evidence suggests that emotional well-being and the feeling of happiness can be associated with gut microbiota profiles in healthy adults, especially when stratified by enterotype. The enterotype-specific links between emotional well-being and gut microbiome diversity suggest that enterotypes may work as an individually tailored intervention. With the expanding interest in the role of the gut microbiome–brain axis in mental health, this nascent field still needs to build up empirical evidence to fill the gap in our understanding of how gut microbiota communicates with the brain to affect emotional well-being and the feeling of happiness.

### Prebiotics, Probiotics, Synbiotics, Postbiotics, and Psychobiotics

#### Probiotics

Probiotics are defined by the International Scientific Association for Probiotics and Prebiotics (ISAPP) as live microorganisms that, when administered in adequate amounts, confer a health benefit on the host.^93,456^ Probiotics usually comprise bacteria, including, among others, *Lactobacillus*, *Bifidobacterium*, and *Bacillus*, although a few strains of the yeast *Saccharomyces* have also been included in probiotic cultures. Probiotic microorganisms must have several features, including a demonstrated benefit to the host and the recognition that they are safe for human consumption, i.e., they have generally been recognized as a safe designation. They are resistant to acid and bile salts that are encountered in the GI tract. The ability to adhere to the intestinal epithelium is a useful trait that promotes both the persistence of the probiotic and host interactions. Other useful features are that they are easily cultured and are resistant to drying, freezing, and freeze-drying so that the probiotic may be produced and stored in bulk. Probiotics can enhance immune function, promote fiber assimilation for SCFA production, and help suppress pathogens in the GI tract.^93,457,458^

#### Prebiotics

Prebiotics are defined by ISAPP as a substrate that is selectively utilized by host microorganisms conferring a health benefit.^31,93^ Therefore, such a definition expands beyond the classical prebiotics composed of polysaccharide carbohydrates and could include any other nondigestible microbiome-fermentable ingredients, such as herbal secondary metabolites and flavobiotics. The definition stresses that the effects must be microbiota-mediated and that the beneficial health effects must be documented for a substance to be considered a prebiotic.^459^ The most well-known products of microbiome-derived fermentation are SCFAs, such as butyric acid, acetic acid, or propionic acid, which have a beneficial effect on the host. Moreover, prebiotics modulate lipid metabolism, increase calcium absorption, have a positive effect on the immune system, and reduce the risk of broad diseases.^458^

#### Synbiotics

ISAPP defined synbiotic as a mixture comprising live microorganisms and substrate(s) selectively used by host microorganisms that confer a health benefit on the host. The panel concluded that defining synbiotics as simply mixtures of probiotics and prebiotics could suppress the innovation of synbiotics that are designed to function cooperatively. The ISAPP panel differentiated synbiotics into two groups: complementary synbiotics and synergistic synbiotics. A complementary synbiotic that has not been designed so that its component parts function cooperatively must be composed of a probiotic plus a prebiotic. A synergistic synbiotic is a synbiotic for which the substrate is designed to be selectively utilized by the coadministered microorganisms.^460^

#### Postbiotics

Postbiotics are a class of products that has emerged in the last 10 years. Postbiotics are defined by the ISAPP as preparations of inanimate microorganisms and/or their components that confer a health benefit on the host. Effective postbiotics must contain inactivated microbial cells or cell components, with or without metabolites, that contribute to observed health benefits.^458,461^ Postbiotics, because they are derived from probiotic microorganisms, have many of the same benefits as probiotics. The effects of postbiotics are often more reliable and predictable than probiotics. Postbiotics tend to have a longer shelf life than probiotics and they are more target-specific and safer in terms of their interaction with the human GI tract.^93^

#### Psychobiotics

The term psychobiotic was coined to describe bacteria that confer mental health benefits. Psychobiotics have demonstrated the ability to improve mood, reduce anxiety, and enhance cognitive function in both healthy populations and patient groups. While the term psychobiotics originally referred to beneficial live organisms, such as bacteria that are specifically beneficial for mental health,^409^ the definition has been expanded in recent years to include prebiotics whose effect on the brain is bacteria-mediated.^462^ It is also worthwhile considering a wider definition of psychobiotics to include any substance that exerts a microbiome-mediated psychological effect or at least possesses psychobiotic properties, such as probiotics, prebiotics, synbiotics, and postbiotics.^462,463^ Recently a new term “phyto-psychobiotics” has been coined to describe medicinal plants whose mental effects are mediated via gut microbiota modulation by prebiotic-like effects, postbiotic-like effects mediated by the active secondary metabolites produced by the gut microbiome from the nondigestible herbal ingredients, or even by antibiotic-like effects as in the case with some medicinal herbs that have a mental impact by reducing the level of pathogenic bacteria.^464^

### Pre-, Pro-, Postbiotics, and Fecal Microbiota Transplantation in the Development Pipelines

**Figure 14:**
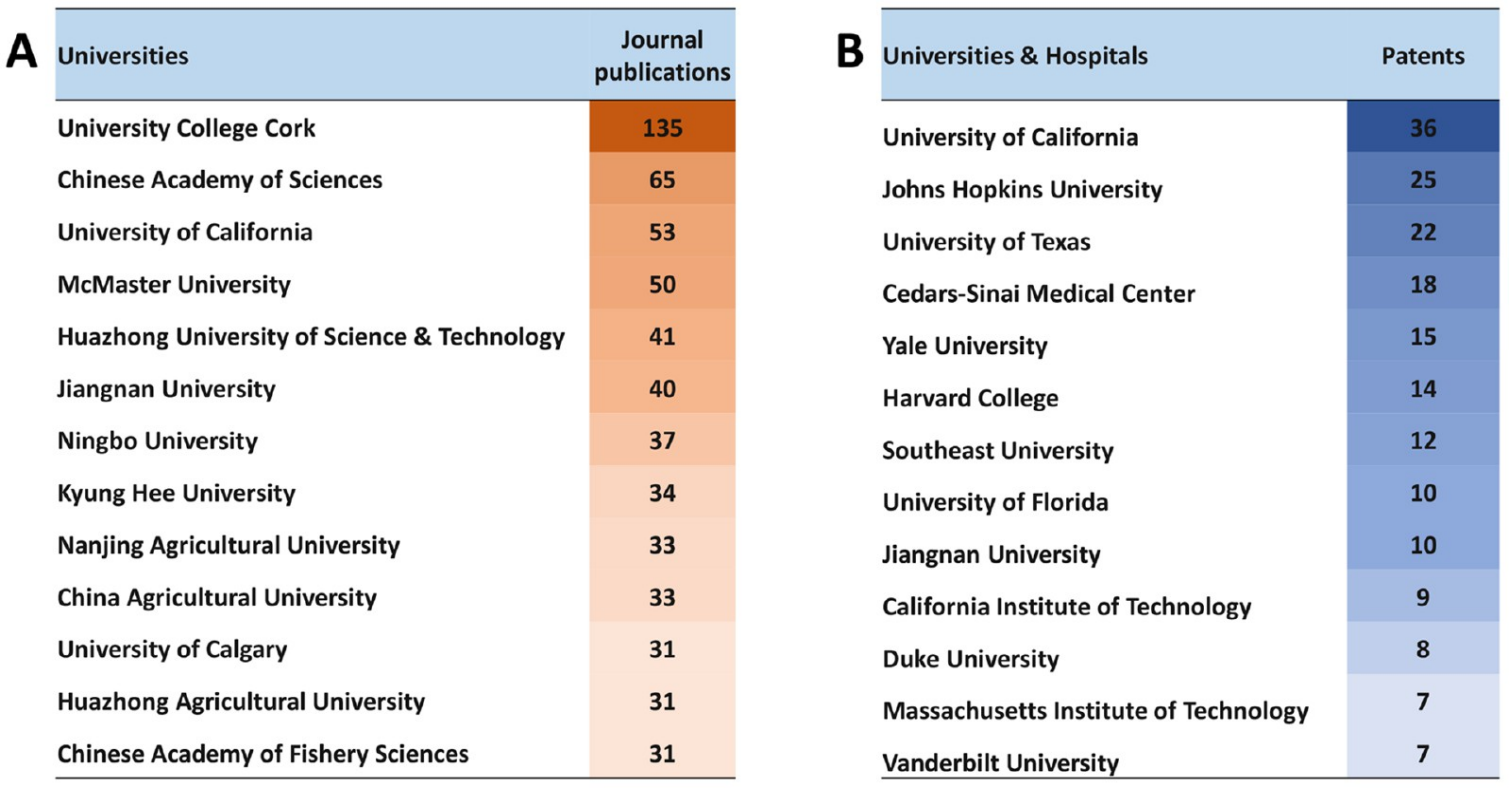
Top universities, research institutes, and hospitals publications (1967–2022) related to gut microbiome research in mental and gastrointestinal health: (A) journal publications and (B) patents.

A search of the CAS Content Collection^84^ reveals the top organizations for research and journal publications related to the gut microbiome and mental and digestive health. All these top players are universities and research institutes, and the University College Cork, the Chinese Academy of Science, the University of California, and McMaster University lead the field (Figure 14A). The lead universities and medical centers related to patenting activity for the gut microbiome in mental and gut health include the University of California, Johns Hopkins University, and the University of Texas (Figure 14B).

#### Private Investment

**Figure 15:**
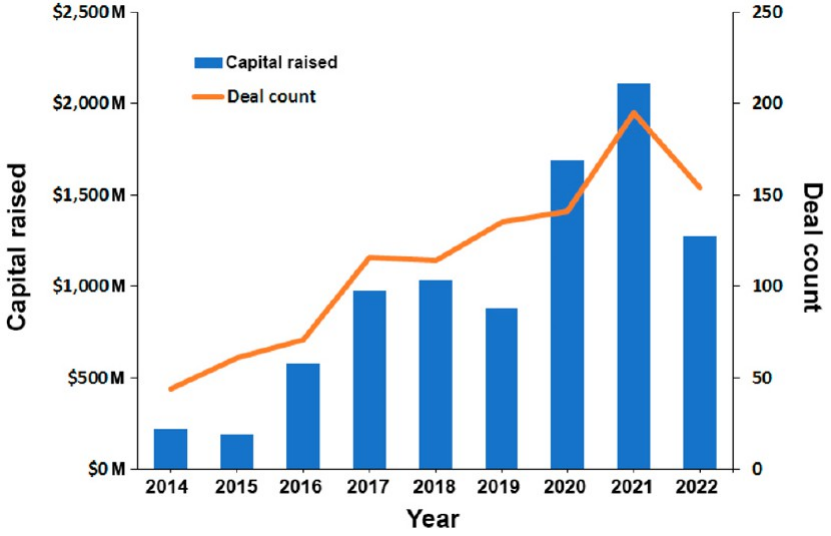
Overall capital raised and deal counts of venture capital investment for the prebiotic, probiotic, and the microbiome field ($) (source: pitchbook.com).

Researching the overall global private investment activities of the microbiome field provides insight into the commercial interest into this area. Performing a search of prebiotics, probiotics, and the microbiome within PitchBook, an online source for investment data, reveals the overall venture capital activities. The search revealed that both capital raised and deal counts from venture capital investment are rising within this industry.^465^ From 2014 through 2018, the total capital raised increased from $250 million to over $1 billion. Deal counts followed the same pattern from 2014 to 2018 and increased from 25 to 125. The number of deal counts in 2019 further increased; however, the overall capital raised fell to just under $900 million. Deal counts continued to rise for 2020 and 2021, along with capital raised, which totaled over $2.1 billion, while 2022 ended with a slight decrease in total venture capital investment (Figure 15). The venture capital investment data in this area clearly shows a recent and increasing commercial interest surrounding prebiotics, probiotics, and the microbiome, thereby revealing its potential promise for therapeutic applications.

### Companies and Academic Institutions Investigating Treatment of Mental Disorders and DGBI through Gut Microbiome Modulation

With the GBA being a bidirectional communication network, we herein examine a highlighted selection of global companies and academic institutions researching and producing prebiotics, probiotics, postbiotics, and utilizing fecal microbiota transplantation to treat a variety of consumer health-related mental disorders and DGBI. This global analysis of worldwide companies and academic institutions, while extensive, is not comprehensive and provides insight into both the present and future state of the field.

#### Probiotics

**Figure 16:**
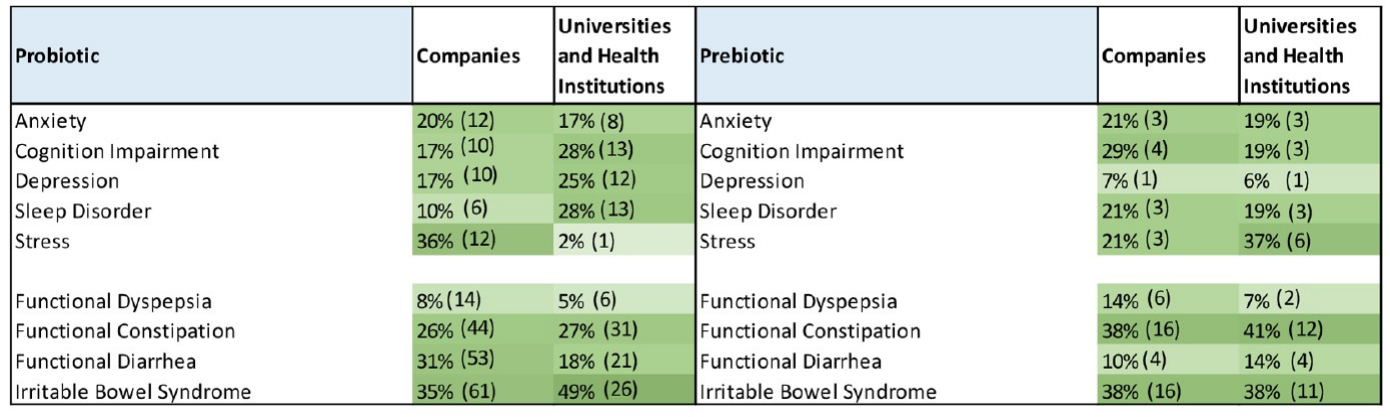
(Left) The percentage and number of analyzed global organizations utilizing probiotics for mental disorders and DGBI treatment. (Right) The percentage and number of analyzed global organizations utilizing prebiotics for mental disorders and DGBI treatment.

Companies, along with universities and health institutions, are utilizing probiotics for the treatment of mental disorders at slightly different rates (Figure 16). Companies are focusing more on stress than universities and medical institutions, with all organizations researching anxiety at about the same rate. Universities and medical institutions are researching depression, cognition impairment, and sleep disorders at a higher rate than industry. With universities and medical institutions on the forefront of research, this shows the up-and-coming possibilities for probiotic products for the treatment of depression, cognition impairment, and sleep disorders being produced for consumers in the future. Research in the area of utilizing probiotics for DGBI is much more extensive and historically established (Supplemental Table 1) than mental health disorders (Figure 16). Companies, universities, and medical institutions research most DGBI at a similar rate, with universities and medical institutions having a higher focus on IBS. IBS is more prevalent among those who eat a Western diet. With more of the world’s population adopting a Western diet, the prevalence of IBS is expected to increase.^466^ The research among universities and medical institutions is shadowing this trend as researchers evaluate probiotic treatment options for this DGBI increasing in prevalence (Figure 16).

#### Prebiotics

Fewer organizations are researching prebiotics for the treatment of mental disorders and DGBI when compared with probiotics (Figure 16). Prebiotics for DGBI are researched more than mental disorders, with more focus on IBS and functional constipation (Figure 16). This field has many future growth opportunities for both industry and research as it grows and becomes established. Similar to the number of organizations, the number of documents in the CAS Content Collections related to prebiotics is about three times lower compared with those related to probiotics. However, the growth rate in the prebiotics research has increased in the last two years.

#### Postbiotics

Postbiotics are the least researched therapeutic examined and are mainly limited to a small commercial presence (Figure 16). With such a small commercial and research presence, the opportunities are also abundant for postbiotic therapeutics.

#### Fecal Microbiota Transplantation

**Figure 17:**
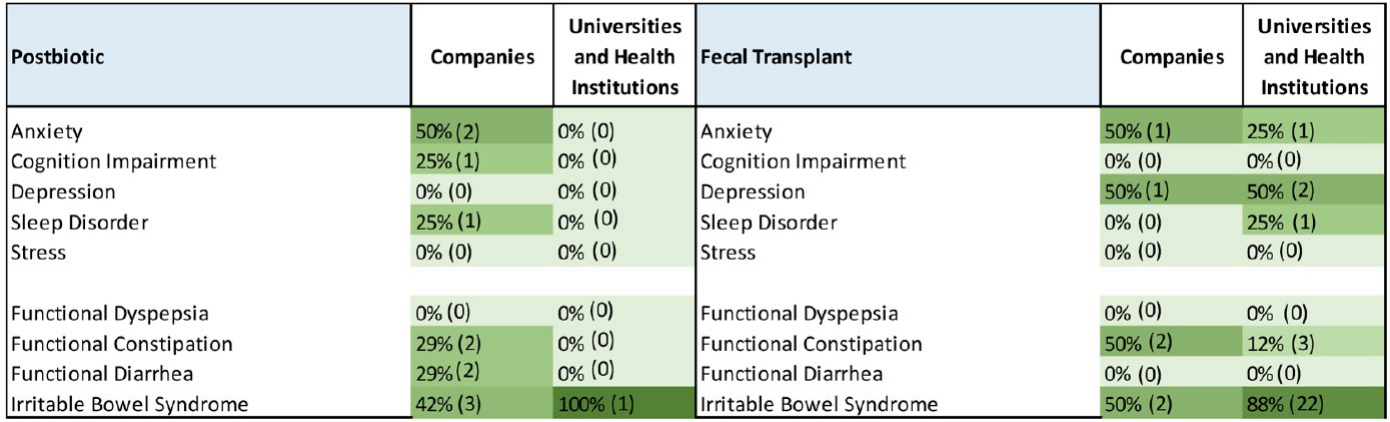
(Left) The percentage and number of analyzed global organizations utilizing postbiotics for mental disorders and DGBI treatment. (Right) The percentage and number of analyzed global organizations utilizing fecal transplant for mental disorders and DGBI treatment.

FMT research is found to be rare in comparison with other microbiome modulation strategies in mental disorders and newly established with only a very small number of companies and universities participating in this field (Figure 17). Most of the FMT research in DGBI focuses on IBS treatment (Figure 17). This field is currently showing clinical scientific evidence for the successful treatment of the serious and sometimes deadly condition of *Clostridium difficile* (C. Diff) infection.^467^ Recently, Australia’s Therapeutic Goods Administration gave the world’s first regulatory approval to company BiomeBank for its biologic microbiome product called Biomictra for the treatment of C. Diff infection.^468^ The US FDA followed a few weeks later with its official approval for the biologic drug consisting of live human-derived fecal microbiota, RBX2600 (Rebyota), produced by Ferring Pharmaceuticals, which is also for the treatment of C. Diff infection.^469^ These first regulatory approvals open this method to endless opportunities for the treatment of many different diseases, and more studies are needed to prove its safety and efficacy.

### Clinical Trials Landscape for Probiotics in Mental Disorders and DGBI

When examining clinical trials utilizing probiotics for the treatment of the mental disorders discussed within, there are currently a total of 52 clinical trials covering all stages between 2004 and 2022 listed on the US NIH clinical trials website.^470^ The most studied mental health disorders are stress, followed by depression, anxiety, cognition impairment, and sleep disorders. Clinical trials utilizing probiotics for the treatment of DGBI are the most prolific and historically studied with 174 clinical trials.^470^ The most studied DGBI are IBS followed by functional constipation, functional diarrhea, and functional dyspepsia.

The most researched probiotics for the treatment of mental disorders are the *Lactobacillus* species and a combination of *Lactobacillus* and *Bifidobacterium* species (Supplemental Table 1). For DGBI, the most widely used probiotic is the *Lactobacillus* species followed by the *Bifidobacterium* species, a combination of *Lactobacillus* and *Bifidobacterium* species, and finally the *Saccharomyces* species^471^ (Supplemental Table 1).

Highlighted clinical trials examining probiotics as a treatment option for mental disorders and DGBI are explored in Tables 4 and 5. A select few are also examined in further detail below to showcase a variety of interventions and targeted conditions in clinical development, along with their status. While experimental data in this area is showing promising results, there are still conflicting results being reported.

**Table 4:** Highlighted Clinical Trials Utilizing Probiotics for the Treatment of Mental Health Disorders

clinical trial identifier	condition	intervention	status
NCT05564767^472^	depression, anxiety, stress	*Bifidobacterium adolescentis* Bif-038, *Lacticaseibacillus rhamnosus* LGG, *Bifidobacterium* BB-12	recruiting
NCT03494725^477^	stress, anxiety	*Lacticaseibacillus paracasei* Lpc-37	complete
NCT04767997^474^	sleep disorder	undisclosed probiotic formulation	recruiting
NCT03601559^478^	cognitive impairment	*Lactobacillus paracasei* Lpc-37	complete
NCT03615651^479^	stress, cognition impairment	*Lactobacillus helveticus*, *Bifidobacterium longum*, *Lactiplantibacillus plantarum*	complete
NCT05567653^473^	stress	*Lactobacillus helveticus* Rosell-52, *Bifidobacterium longum* Rosell-175	recruiting
NCT03370458^480^	stress	*Lactobacillus plantarum* DR7	complete

**Table 5:** Highlighted Clinical Trials Utilizing Probiotics for the Treatment of DGBI

clinical trial identifier	condition	probiotic intervention	status
NCT02592200^485^	functional constipation	*Lactobacillus gasseri* DSM 27123	complete
NCT04304170^486^	functional constipation	*Bifidobacterium animalis lactis* (LMG P-28145)	complete
NCT04662957^487^	diarrhea-predominant irritable bowel syndrome	*Bifidobacterium breve* BB010, *Bifidobacterium longum* BL020, *Bifidobacterium bifidum* BF030, *Bifidobacterium lactis* BL040, *Lactobacillus rhamnosus* LR110, *Lactobacillus paracasei* LPC100, *Lactobacillus acidophilus* LA120, *Lactobacillus casei* LC130, *Lactobacillus plantarum* LP140, *Streptococcus thermophilus* ST25	complete
NCT05566171^488^	functional constipation	*Bifidobacterium lactis* CNCM I-2494, *Bifidobacterium lactis* DN 173-010	enrolling by invitation
NCT01463293^489^	functional constipation	*Bifidobacterium lactis* HN019	complete
NCT01102036^490^	functional constipation	*Lactobacillus paracasei* F19, *Lactobacillus paracasei* LA-5, *Bifidobacterium lactis* BB-12	complete
NCT03721107^491^	diarrhea- and constipation-predominant irritable bowel syndrome	*Blautia hydrogenotrophica*	complete
NCT00534170^492^	functional diarrhea	*Lactobacillus casei* Shirota, *Bifidobacterium breve* Yakult	complete
NCT00794924^493^	functional diarrhea, functional constipation	*Streptococcus thermophilus*, *Bifidobacterium breve*, *Bifidobacterium longum*, *Bifidobacterium infantis*, *Lactobacillus acidophilus*, *Lactobacillus plantarum*, *Lactobacillus paracasei*, *Lactobacillus delbrueckii* subsp. *bulgaricus*	complete
NCT02213172^494^	irritable bowel syndrome	*Bifidobacterium longum* R0175, *Lactobacillus paracasei* HA-196	complete
NCT00807326^495^	functional diarrhea	*Saccharomyces boulardii*	complete
NCT05054309^496^	irritable bowel syndrome	*Bifidobacterium longum* NCC3001	recruiting
NCT01099696^497^	functional dyspepsia	*Bifidobacterium infantis* 35624	complete
NCT01887834^498^	irritable bowel syndrome	*Lactobacillus gasseri*, *Bifidobacterium bifidum*, *Bifidobacterium longum*	complete
NCT04605783^499^	functional diarrhea	*Saccharomyces boulardii* CNCM I-745	not yet recruiting
NCT04950296^500^	irritable bowel syndrome with diarrhea	*Lactobacillus plantarum* UALp-05	complete
NCT05149599^501^	irritable bowel syndrome	*Saccharomyces cerevisiae*	complete

#### Mental Disorders

Clinical trial number NCT05564767 is currently recruiting participants to assess the treatment of depression and anxiety in adults by utilizing probiotics *Bifidobacterium* alone and *Lacticaseibacillus* combined with *Bifidobacterium*.^472^ Clinical trial number NCT05567653 is also recruiting for its study researching the treatment of stress in dancers with a probiotic product combination including *Lactobacillus* and *Bifidobacterium*.^473^ Another study (NCT04767997) looking at the effects of probiotics on sleep disorders is also currently recruiting subjects.^474^ Clinical trial number NCT03615651 researched the effect of a probiotic mixture containing *Lactobacillus helveticus*, *Bifidobacterium longum*, and *Lactiplantibacillus plantarum* on subjects’ functional brain responses during an emotionally stressful attention task.^472^ Their findings showed a positive effect on brain responses in regions implicated in emotional and cognition processing, which supports the growing evidence that probiotics can help positively influence emotional regulation and brain function.^475^ Another study (NCT04767997) that is looking at the effects of probiotics on sleep disorders is also currently recruiting subjects. Finally, a recently completed study (NCT03494725) found that supplementation with probiotic *Lacticaseibacillus paracasei* Lpc-37 significantly reduced perceived stress and anxiety in study participants.^476^

#### DGBI

Clinical trial number NCT05566171 is currently recruiting participants to research the treatment of functional constipation with two *Bifidobacterium* strains. *Bifidobacterium* was also researched in clinical trial number NCT04304170 and NCT01463293 for the treatment of functional constipation. *Lactobaccillus* and a combination of *Bifidobacterium* species and *Lactobacillus* were also researched for the treatment of functional constipation in completed clinical trials NCT01102036 and NCT02592200, respectively. Utilizing a combination of *Streptococcus*, *Bifidobacterium*, and *Lactobacillus* species, clinical trial number NCT00794924 researched the treatment of functional constipation and diarrhea in elderly hospitalized patients. The study showed positive results with the probiotics reducing days patients suffered from diarrhea or received laxatives, thereby displaying a positive effect on bowel movements. With diarrhea remaining a major public health concern in developing countries, study NCT00534170 researched the use of a probiotic drink containing both *Lactobacillus* and *Bifidobacterium* species for the treatment of diarrhea in young children. The study revealed the ingestion of a daily probiotic drink could prevent diarrhea in young children in a community setting within a developing country.^481^ Another study (NCT00807326) also researched the treatment of functional diarrhea. They compared the treatment results of antigas/antidiarrheal drug combination loperamide/simeticone and probiotic yeast *Saccharomyces boulardii*. They discovered that while the probiotic did help alleviate symptoms, it was inferior to loperamide/simeticone.^482^ Clinical trial number NCT01099696 also had discouraging results researching the treatment of functional dyspepsia with probiotic *Bifidobacterium infantis* 35624. While previous studies showed promise in patients with IBS, *Bifidobacterium infantis* 35624 did not show significant improvement in symptoms of abdominal discomfort and bloating in that study’s participants. Finally, a study (NCT04662957) researching a multistrain probiotic mixture of four *Bifidobacterium*, five *Lactobacillus*, and one *Streptococcus* species concluded its probiotic could offer benefits for patients with diarrhea-predominant IBS.^483^ Another study (NCT03721107) showed more positive results for the treatment of both diarrhea and constipation-predominant IBS. Patients reported improvement in bowel symptoms and pain with the use of *Blautia hydrogenotrophica*.^484^

#### Clinical Trials Landscape for Prebiotics in Mental Disorders and DGBI

When examining clinical trials utilizing prebiotics for treatment of the mental disorders discussed within, there are currently a total of 15 clinical trials.^470^ The most studied mental health disorder is stress, followed by anxiety, depression, sleep disorders, and cognition impairment.^470^ A total of 50 clinical trials utilize prebiotics for the treatment of DGBI.^470^ Similar to probiotics, the most studied DGBI using prebiotics is IBS, followed by functional constipation, functional diarrhea, and functional dyspepsia.^470^ The most commonly used prebiotic in clinical trials is galacto-oligosaccharides (GOS), followed by other sugars such as fructan, glucan, and dextrose, along with various fibers.

Highlighted clinical trials examining the treatment of mental disorders and DGBI with prebiotics are explored in Tables 6 and 7. A select few are also examined in further detail below to showcase the variety of interventions and targeted conditions, along with their status in clinical development. The use of prebiotics for the relief of mental and gastrointestinal disorder symptoms is producing promising results.

**Table 6:** Highlighted Clinical Trials Utilizing Prebiotics for the Treatment of Mental Health Disorders

clinical trial identifier	condition	intervention	status
NCT05372601^504^	stress	GOS	complete
NCT05239845^505^	sleep disorder	polydextrose, GOS	recruiting
NCT04324749^506^	cognitive impairment, stress	roasted peanuts, peanut butter	complete
NCT05528575^507^	stress	GOS, inulin, resistant potato starch RS2	active
NCT04616937^508^	anxiety, cognitive impairment	GOS	complete

**Table 7:** Highlighted Clinical Trials Utilizing Prebiotics for the Treatment of DGBI

clinical trial identifier	condition	intervention	status
ISRCTN54052375^512^	irritable bowel syndrome	GOS	complete
NCT04491734^513^	gastresophageal reflux	maltosyl-isomaltooligosaccharides (MIMO)	complete
NCT05207618^514^	irritable bowel syndrome with diarrhea	chestnut and quebracho tannin extract	complete
ACTRN12612001270808^510^	functional constipation	green kiwi prebiotic, gold kiwi prebiotic	complete
NCT05340712^515^	functional constipation	infant formula with lactose (prebiotic) along with probiotics	recruiting

##### Mental Disorders

Clinical study NCT04616937 researches the use of GOS for the treatment of anxiety by altering the gut microbiota. GOS increases probiotic bacteria *Bifidobacterium* abundance in the gut microbiome. The study reports that the supplementation of GOS may improve signs of anxiety and cognition impairment with an increase of reported attention.^502^ Another recent study (NCT04324749) researched the prebiotic effect of peanut and peanut butter consumption on the cognitive and stress response of college students. The peanut prebiotic fiber and polyphenol content appears to enhance memory function and reduce stress because of the presence of both short-chain and very long-chain saturated fatty acids for healthy young subjects.^503^

##### DGBI

A published case study from 2019 showed a new observation that some patients taking a specific prebiotic soluble fiber, maltosyl-isomaltooligosaccharide (MIMO), had their gastroesophageal reflux symptoms resolve.^509^ Clinical trial NCT04491734 followed about a year later to research this effect. The prebiotic MIMO reduced the severity and frequency of gastroesophageal reflux symptoms and improved the quality of life for participants. Clinical study ACTRN12612001270808 researched the use of a green kiwi prebiotic (inulin) and a gold kiwi prebiotic to treat functional constipation. The study showed a successful increase in bowel movements and also revealed that green kiwi prebiotic (inulin) supports the increase of two probiotic bacteria, *Bifidobacteria* and *Lactobacillus*, within the gut microbiome.^510^ Another research study shows that gold kiwi prebiotic increased the abundance of *Faecalibacterium prausnitzii* within the gut microbiome, as well.^511^*F. prausnitzii* is a butyrate producer and displays anti-inflammatory effects.^511^ When prebiotic GOS was tested in a clinical trial (ISRCTN54052375), it increased the probiotic bacteria *Bifidobacteria* within the gut. This helped alleviate symptoms of IBS, thereby showing that GOS is a potential therapeutic agent for this disorder.^512^

### Clinical Trials Landscape for Postbiotics and FMT in Mental Disorders and DGBI

Postbiotic and FMT are the least researched among all clinical trials explored. When examining clinical trials utilizing postbiotics for the treatment of mental disorders, anxiety is the only disorder studied (Supplemental Table 1). Clinical trials utilizing postbiotics for the treatment of DGBI focus on IBS.^470^ Only three clinical trials are researching FMT for the mental health disorders of depression, anxiety, and sleep disorders. Fecal microbiota transplants researching DGBI is higher with 27 trials researching both IBS and constipation.

Highlighted clinical trials examining the treatment of mental disorders and DGBI with postbiotics and FMT are explored in Tables 8 and 9. A select few are also examined in further detail below to showcase a variety of interventions and targeted conditions, along with their status in clinical development.

**Table 8:** Highlighted Clinical Trials Utilizing Postbiotics for the Treatment of Mental Disorders and DGBI

clinical trial identifier	condition	intervention	status
NCT05475314 (517)	irritable bowel syndrome	microbially fermented postbiotic oat drink	complete
NCT05562739 (518)	anxiety	multistrain postbiotic	not yet recruiting
NCT05339243 (519)	irritable bowel syndrome with diarrhea	heat-treated *Bifidobacterium longum* ES1	recruiting

**Table 9:** Highlighted Clinical Trials Utilizing Fecal Microbiota Transplantation for the Treatment of Mental Disorders and DGBI

clinical trial identifier	condition	intervention	status
NCT03822299^522^	irritable bowel syndrome	fecal microbiota transplantation	complete
NCT02092402^523^	irritable bowel syndrome	fecal microbiota transplantation	complete
NCT05035784^524^	functional constipation	fecal microbiota transplantation	recruiting
NCT05427331^525^	chronic insomnia	fecal microbiota transplantation capsule	recruiting

### Postbiotic Clinical Trials Landscape for the Treatment of Mental Disorders and DGBI

Clinical study NCT05562739 (recruiting) is researching a multistrain postbiotic for anxiety treatment in individuals who were placebo nonresponders from part one of the trial. A recently completed study (NCT05475314) researched the use of a postbiotic fermented oat drink for the treatment of IBS. The study resulted in positive outcomes and showed symptom relief in IBS subjects.^516^ Lastly, clinical trial NCT05339243 is currently recruiting for their study investigating both *Bifidobacterium longum* ES1 and the postbiotic heat-treated *Bifidobacterium longum* ES1 for the treatment of IBS symptoms in subjects with diarrhea-predominant IBS.

### FMT Clinical Trials Landscape for the Treatment of Mental Disorders and DGBI

Clinical trial NCT05427331 is currently recruiting for its study examining sleep disorders. FMT through oral capsule administration will be researched to access sleep improvement in patients with insomnia. While fecal transplants are less prevalent for mental disorders, they have shown promise for DGBI with clinical trials by showing favorable results for the treatment of IBS. Clinical trial NCT02092402 showed effective treatment for patients with IBS when using a donor with a diverse microbial gut composition.^520^ Another study (NCT03822299) also showed success for FMT in IBS. Just as with trial NCT02092402, it also found that the donor’s fecal composition with a favorable microbial signature and diverse gut microbiota was essential for successful treatment.^123^ Three years after treatment, the study was still experiencing high response rates and long-standing effects.^521^

#### Noteworthy Probiotic and Prebiotic Patents

There are a diverse and growing number of patents related to probiotics and prebiotics in the CAS Content Collection. Listed in Table 10 are noteworthy prebiotic and probiotic patents related to the treatment of mental disorders and DGBI.

**Table 10:** Notable Prebiotic, Probiotic, and Postbiotic Patents

patent number	title	summary
WO2016085356A1	gold kiwifruit compositions and methods of preparation and use thereof	Prebiotic compositions prepared from gold varieties of *Actinidia chinensis*. These prebiotic compositions treat or prevent DGBI, such as constipation, and IBS.
WO2022191767A1	GOS preconditioning *L. reuteri* and GOS in final formulation	Enhancing the survival and activity of probiotic *Lactobacillus reuteri* strains by preconditioning *L. reuteri* with GOS. This method produces high synbiotic and beneficial effects of the probiotic bacteria in the gastrointestinal tract, such as boosting calcium and iron solubility, along with enhancing the production of lactic and acetic acid.
US20160058808A1	microbe-based modulation of serotonin biosynthesis	Methods and probiotic compositions that can be used to modulate serotonin levels and adjust the composition of gut microbiota along with adjusting the level of serotonin-related metabolites.
WO2022182908A1	probiotic therapies for social deficit and stress response	Bacterial species, including probiotic *Enterococcus faecalis*, for use in the treatment of social behavioral deficit symptoms, such as depression, by increasing social behavior and decreasing corticosterone levels along with c-Fos expression in the brain.
US9192618B2	method of treating constipation-predominant irritable bowel syndrome	Prebiotic or probiotic agent that inhibits the growth of methanogenic bacteria or promotes the growth of competing intestinal microbiotia for the treatment of constipation predominant IBS.
US10022408B2	probiotic *Bifidobacterium adolescentis* strains	Novel isolated strains of probiotic *Bifidobacterium adolescentis* for the prevention, alleviation of symptoms, or treatment of intestinal inflammatory conditions, such as IBS.
WO2005003329A1	novel GOS composition and the preparation thereof	Novel strains of *Bifidobacterium hifidum* capable of producing a novel galactosidase enzyme activity that converts lactose to a novel mixture of GOS. The mixture of prebiotic oligosaccharides improves gut health by promoting the growth of bifidobacteria in the gut.
US20220040242A1	modulation of the gut microbiome to treat mental disorders or diseases of the central nervous system	Methods of treating at least one symptom of a mental disorder and the central nervous system by modulating the amount of GABA produced in the gut. Also disclosed are methods of identifying and creating probiotic bacterial strains capable of producing GABA.
WO2016029198A1	process for the production of isomaltooligosaccharide	Provides a method for the production of prebiotic oligosaccharides by the fermentation of dextransucrase-producing microorganisms.
WO2022214700A1	*Lacticaseibacillus paracasei* EM025-11 and uses thereof	A probiotic strain of *Lacticaseibacillus paracasei* EM025-11 that adheres to intestinal epithelial cells and has anti-inflammatory activity by upregulating genes associated with immune engagement for the treatment of IBS with constipation.
US20220280576A1	*Bifidobacterium longum* and functional GI disorders	Methods for treating functional GI disorders with probiotic *Bifidobacterium longum* ATCC BAA-999.
WO2019149941A1	postbiotic-based composition for the modulation of immune system activation and protection of mucosal barriers	A fermented supernatant of *Lactobacillus casei* or *paracasei* species for the promotion of human health and prevention of inflammatory disorders. The postbiotic was shown to stimulate peripheral blood mononuclear cells and protect from endotoxic shock and *Salmonella* infection.
US8551498B2	solid composition containing *Bacillus*-type nonpathogenic bacterial spores	Composition of spores of probiotic bacteria *Bacillus* useful in the pharmaceutical, veterinary, and nutrition fields.
ES2824536T3	use of microbial communities for human and animal health	Mixture of probiotic bacteria belonging to at least six or seven bacterial species to prevent or treat GI disorders.
US20220233559A1	xylooligosaccharide as a multifunctional prebiotic	Prebiotic mixture of xylooligosaccharides derived from sugar cane that were shown to modulate the levels of probiotic bacteria *Bifidobacteria* and *Lactobacillus* in the microbiome.
WO2022173764A1	nutritional plant-based foods and beverages, methods of manufacture, and methods of treatment	Formulation of a prebiotic beverage whose ingestion modulates the gut microbiome by enhancing the growth of *Bifidobacterium*, improving the production of SCFAs, and reducing the levels *Escherichia coli.*
US20220315960A1	method for producing gamma-aminobutyric acid and fermented culture prepared thereby	Process to produce gamma-aminobutyric acid from glutamic acid and a probiotic composition capable of this biotransformation. Ideally, the composition contains different probiotic bacteria *Bifidobacterium* and *Lactobacillus* strains.
WO2022208458A1	inactivated strains of bacteria, such as viable but nonculturable bacteria, compositions, and use thereof	Postbiotic composition of different gamma-irradiated members of bacterial species *Lactobacillus*, *Lacticaseibacillus*, *Bifidobacterium*, and *Lactiplantibacillus* to treat several gastrointestinal disorders.
EP3932415A1	gut microbiota composition and uses thereof	Probiotic composition for the prevention and/or treatment of a mental disorder with memory impairment by gut microbiome modulation for an increase in memory scores.

## Conclusions and Perspective

Gut microbiota in humans evolving throughout life has been demonstrated to play a key role in health and disease. In healthy individuals, the intestinal microbiota has a multitude of beneficial functions, including metabolic energy utilization, protection from pathogenic attack, and immunomodulation. Furthermore, it is becoming increasingly documented that bidirectional signaling takes place between the gut and the brain and involves gut microbiota. This relationship encompasses various pathways, such as the vagus nerve; the hypothalamic–pituitary–adrenal axis; and immune, hormonal, and metabolic pathways to control various aspects of homeostasis, including the appropriate development and maintenance of digestive and mental functions. A dysbiosis of the intestinal microbiota is becoming documented as a factor in the pathogenesis of various pathological conditions, including a plethora of mental, metabolic, and digestive disorders. The diverse etiology of these disorders has been related to different microbes, although insufficient information is currently available on the causal direction of the association. Recent impressive advances in next generation sequencing technologies, along with the progress and innovations of metagenomics, metabolomics, multiomics, bioinformatics, and artificial intelligence tools, have provided prospects to better characterize the microbial populations and their functions and help in better correlation prediction. Moreover, studies using germ-free animals have provided important knowledge on causality rather than association. The research focus needs to be further shifted from individual microbes and their role in influencing health and disease toward the gut microbiome ecosystem. A better understanding of fundamental rules driving interactions within gut microbial communities and the dynamics through which they are acquired, transmitted, and adapted to single individuals is needed to make further progress.

The gut microbiome–brain axis embodies a sophisticated network of biological constructs that scientists are only beginning to understand. The nutritional and therapeutic approaches to modulate this axis are ultimately aimed at improving human quality of life. Some products are even already on the market, including foods and supplements that promise to improve gut, mood, sleep, or cognitive performance. The scientific background behind some of these promises is, however, still arguable or unknown.

The gut microbiome offers interesting possibilities to enhance therapies. Future studies should focus on identifying if gut microbiome signatures correlate with fecal/luminal metabolites and/or cytokines that might translate into marked differences in patients’ life. Moreover, more studies evaluating the potential of therapeutic modulation of the microbiome by biotics/fecal transplant to enhance therapeutic options for diseases are needed. Nowadays, research has shown that fecal transplants can restore healthy bacteria in the lower intestine, which can help control diseases caused by pathogenic bacteria. As a result, the first approved fecal transplant drugs are already a fact. Australia’s Therapeutic Goods Administration was the first to grant approval to biotechnology company BiomeBank for its microbiome-based therapy product Biomictra for treating infections from *Clostridioides difficile* bacteria.^526,527^ Days later, the US FDA also approved its first fecal microbiota product Rebyota produced by Ferring Pharmaceuticals for the prevention of recurrence of *C. difficile* infection in adults.^528^

The multitude of relations between the microbiota, gut, and brain are now well-documented. The next step—moving on from correlative analysis toward understanding the mechanisms behind these relations and identifying the best ways to adapt and adjust the microbiota for potential therapeutic approaches—is now the required pathway. Some of the key setbacks in existing knowledge include understanding the immunological function of specific microbes in the human gut microbiota and their role in neurodegenerative and psychiatric disorders and how microbial metabolites influence brain function in tandem with immunological and neurological signaling molecules.

Despite the advances in microbiome knowledge, a lack of standardization significantly complicates and obstructs comparisons across studies, thus hampering insights into the structure and function of microbial populations. Recent efforts in the introduction of standardized protocols and analytical methods to characterize microbiota and explore the relationships and co-occurrences of microbially related metabolites and microbial taxa^529^ would allow for great improvements in examinations of microbial diversity.

One of the significant challenges in microbiota-based medicine is the delineation of healthy microbiota. Variations in microbiota composition between individuals can be large, i.e., microbiota turn out to be pretty much person-specific, which greatly complicates a “one size fits all” strategy in targeting it. However, it also offers chances since the microbiota might be the outlet for an effective future personalized medicine approach.^530^ Overall, clinical studies are hampered by the deficiency of specific biomarkers. Yet, recent meta-analyses have validated a positive assessment of the use of psychobiotic interventions for anxiety, schizophrenia, or cognitive performance, which aim at the diversity and complexity of gut microbiota, as well as the various confounding factors that may affect it.^462,531−536^

Although the prevention of brain disorders still remains out of reach, the knowledge of healthy microbiota and their communication pathways could enable an early prediction of such disorders. Thus, the first signs of neurodegenerative conditions, such as Alzheimer’s and Parkinson’s diseases, are known to develop many years before diagnosis. It might become thinkable to slow down neurodegenerative processes by altering the microbiome. Such possibilities have inspired a growing number of scientists to initiate start-up companies examining therapeutics for the treatment of neurological and other disorders through microbiome modulation. Private investors are showing a strong upward trend to fund such clinical research.

Perfecting the gut microbiota through fecal transplants; pro-, pre-, post-, and syn-biotics; healthy diet; and/or healthy lifestyle to control gut micriobiome–brain axis functions and promote mental and digestive health will be a promising field in the future. Patients suffering from mental and/or digestive disorders will get help through such treatments. Healthy individuals will promote their homeostasis and resilience from these remedies.

## Supporting Information Available

Prebiotic, probiotic, postbiotic, and fecal transplant therapeutic clinical trial data investigating the treatment of mental disorders and DGBI through gut microbiome modulation (XLSX)

## Abbreviations

4-EPS: 4-ethylphenylsulfate
5-AVAB: 5-aminovaleric acid betaine
BAs: bile acids
BBB: blood-brain barrier
BCFA: branched-chain fatty acids
CAN: central autonomic network
CEN: central executive network
CNS: central nervous system
DGBI: disorders of gut–brain interaction
DMN: default mode network
EAN: emotional arousal network
ENS: enteric nervous system
FC: functional constipation
FCT: fecal microbiota transplantation
FD: functional dyspepsia
FGIDs: functional gastrointestinal disorders
GBA: gut–brain axis
HC: healthy controls
HPA: hypothalamus–pituitary–adrenal
IBS: irritable bowel syndrome
IHMC: International Human Microbiome Consortium
MPs: muramyl peptides
OCC: occipital network
RDA: recommended daily allowance
SAL: salience network
SCFA: short-chain fatty acids
SMN: sensorimotor network
TMAO: trimethylamine-*N*-oxide
